# The mitochondrial NAD ^+^ transporter (NDT1) plays important roles in cellular NAD ^+^ homeostasis in *Arabidopsis thaliana*

**DOI:** 10.1111/tpj.14452

**Published:** 2019-08-09

**Authors:** Izabel de Souza Chaves, Elias Feitosa-Araújo, Alexandra Florian, David B. Medeiros, Paula da Fonseca‐Pereira, Lennart Charton, Elmien Heyneke, Jorge A.C. Apfata, Marcel V. Pires, Tabea Mettler‐Altmann, Wagner L. Araújo, H. Ekkehard Neuhaus, Ferdinando Palmieri, Toshihiro Obata, Andreas P.M. Weber, Nicole Linka, Alisdair R. Fernie, Adriano Nunes‐Nesi

**Affiliations:** ^1^ Max Planck Partner Group Departamento de Biologia Vegetal Universidade Federal de Viçosa 36570‐900 Viçosa Minas Gerais Brazil; ^2^ Max‐Planck‐Institute of Molecular Plant Physiology Am Mühlenberg 1 14476 Potsdam‐Golm Germany; ^3^ Department of Plant Biochemistry Heinrich Heine University Düsseldorf 40225 Düsseldorf Germany; ^4^ Department of Plant Physiology University of Kaiserslautern D‐67663 Kaiserslautern Germany; ^5^ Department of Biosciences, Biotechnology and Biopharmaceutics University of Bari 70125 Bari Italy

**Keywords:** *Arabidopsis thaliana*, nicotinamide adenine dinucleotide, transporter, pollen viability, starch metabolism

## Abstract

Nicotinamide adenine dinucleotide (NAD ^+^) is an essential coenzyme required for all living organisms. In eukaryotic cells, the final step of NAD ^+^ biosynthesis is exclusively cytosolic. Hence, NAD ^+^ must be imported into organelles to support their metabolic functions. Three NAD ^+^ transporters belonging to the mitochondrial carrier family (MCF) have been biochemically characterized in plants. *At*NDT1 (*At*2g47490), focus of the current study, *At* NDT2 (*At*1g25380), targeted to the inner mitochondrial membrane, and *At* PXN (*At*2g39970), located in the peroxisomal membrane. Although *At* NDT1 was presumed to reside in the chloroplast membrane, subcellular localization experiments with green fluorescent protein (GFP) fusions revealed that *At* NDT1 locates exclusively in the mitochondrial membrane in stably transformed Arabidopsis plants. To understand the biological function of *At* NDT1 in Arabidopsis, three transgenic lines containing an antisense construct of *AtNDT1* under the control of the 35S promoter alongside a T‐DNA insertional line were evaluated. Plants with reduced *AtNDT1* expression displayed lower pollen viability, silique length, and higher rate of seed abortion. Furthermore, these plants also exhibited an increased leaf number and leaf area concomitant with higher photosynthetic rates and higher levels of sucrose and starch. Therefore, lower expression of *AtNDT1* was associated with enhanced vegetative growth but severe impairment of the reproductive stage. These results are discussed in the context of the mitochondrial localization of *At* NDT1 and its important role in the cellular NAD ^+^ homeostasis for both metabolic and developmental processes in plants.

## Significance Statement

The mitochondrial NAD^+^ carrier (NDT1) plays an important role in cellular NAD^+^ homeostasis in leaves. Impaired NDT1 function results in reduced pollen grain viability, tube growth and seed filling, seed germination and seedling establishment.

## Introduction

Nicotinamide adenine dinucleotide (NAD^+^) and its phosphorylated derivative (NADP^+^) are central coenzymes implicated in cellular homeostasis. Alteration in the balance of the anabolism and catabolism of these nucleotides does not only affect metabolism but also the redox poise of the entire cell, thereby strongly impacting plant growth and development (Noctor *et al*., 2006; Hashida *et al*., 2009, 2010; Gakière *et al*., 2018). Along with its derivative forms, NAD^+^ participates in several biological reactions in glycolysis, the tricarboxylic acid (TCA) cycle, glycine decarboxylation, the Calvin−Benson cycle, and the β‐oxidation in peroxisomes (Bernhardt *et al*., 2012; Geigenberger and Fernie, 2014).

NAD^+^ is widely used as coenzyme for reductive/oxidative processes, playing important roles in the operation of a range of dehydrogenase activities (Selinski *et al*., 2014). In addition, an important role for NAD^+^ metabolism has been demonstrated in pollen maturation and germination (Hashida *et al*., 2013) and for the energy generation during pollen germination and tube growth (Cárdenas *et al*., 2006; Selinski and Scheibe, 2014). The NADPH generated in heterotrophic plastids by the oxidative pentose phosphate pathway (OPPP) provides the reducing power required for several pathways, such as fatty acid biosynthesis (Neuhaus and Emes, 2000), as well as nitrogen assimilation (Bowsher *et al*., 2007) and amino acid biosynthesis. Moreover, both NAD^+^ and NADP^+^ play an essential role in signalling pathways through their interaction with reactive oxygen species (ROS) (Hashida *et al*., 2010). Consequently, it is assumed that these two coenzymes are involved in the acclimation to environmental stresses such as UV radiation, salinity, temperature, and drought (De Block *et al*., 2005). Over and above this, it has been proposed that pyridine nucleotide metabolism is important for seed germination (Hunt *et al*., 2007), stomatal movement (Hashida *et al*., 2010), bolting (Liu *et al*., 2009), development (Hashida *et al*., 2009), senescence (Schippers *et al*., 2008), and nitrogen assimilation (Takahashi *et al*., 2009).

NAD^+^ biosynthesis in plants occurs via either the *de novo* or the salvage pathway (Noctor *et al*., 2006; Hashida *et al*., 2009). The *de novo* pathway starts in plastids using aspartate or tryptophan as precursors, while the salvage pathway starts with nicotinamide (NAM) or nicotinic acid (NA). Both metabolic fluxes converge in the formation of nicotinic acid mononucleotide (NAMN), which in turn gives rise to NAD^+^. Furthermore NAD^+^ kinases can synthetize NADP^+^ from NAD^+^ and ATP in the cytosol (NADK1; Berrin *et al*., 2005; Waller *et al*., 2010) and in the chloroplasts (NADK2; Chai *et al*., 2005, 2006). In addition, peroxisomal NADH kinase, which uses NADH rather than NAD^+^ as substrate to produce NADPH, has been found in *Arabidopsis thaliana* (NADK3; Turner *et al*., 2005; Waller *et al*., 2010). Since the last step of NAD^+^ synthesis takes place in the cytosol, NAD^+^ must be imported into the cell organelles to allow proper metabolism (Noctor *et al*., 2006).

In yeast, two carrier proteins called *Sc*NDT1 and *Sc*NDT2 (NDT: NAD^+^ transporter), which are capable of transporting NAD^+^, have been identified (Todisco *et al*., 2006). The characterization of *Sc*NDT1 protein revealed its location in the inner mitochondrial membrane and its high NAD^+^ transport activity in exchange with ADP and AMP *in vitro* (Todisco *et al*., 2006). The lack of both *Sc*NDT proteins in yeast assigns a function for supplying NAD^+^ to the mitochondrial matrix (Todisco *et al*., 2006). Three genes encoding proteins capable of NAD^+^ transport have been identified in Arabidopsis. *AtNDT2* is located in the inner mitochondrial membrane (Palmieri *et al*., 2009) and *AtPXN* resides in the peroxisomal membrane (Agrimi *et al*., 2012; Bernhardt *et al*., 2012). Moreover, re‐evaluation of the subcellular localization of *AtNDT1*, previously reported to encode a protein targeted to the inner membrane of the chloroplast (Palmieri *et al*., 2009), revealed the exclusive mitochondrial localization of this carrier (the present work). Interestingly, the two mitochondrial NAD^+^ carrier proteins found in Arabidopsis, *At*NDT1 and *At*NDT2, have similar substrate specificity, importing NAD^+^, but not NADH, nicotinamide, nicotinic acid, NADP^+^ or NADPH, against ADP or AMP (Palmieri *et al*., 2009). In contrast, the *At*PXN transporter has a versatile transport function *in vitro*, also using NADH and CoA as substrates (Agrimi *et al*., 2012; Bernhardt *et al*., 2012). Yeast complementation studies revealed that *At*PXN favours the import of NAD^+^ in exchange for AMP in intact yeast cells (van Roermund *et al*., 2016). Furthermore, expression of either *At*NDT1 or *At*NDT2 is able to complement the phenotype of *S. cerevisiae* cells lacking their NAD^+^ mitochondrial transporters and increase the mitochondrial NAD^+^ content of the double mutant strain devoid of their two NAD^+^ mitochondrial transporters (Palmieri *et al*., 2009). In addition, the expression of either *At*NDT2 or *Sc*NDT1 in human cells increases the NAD^+^ content within the mitochondria (VanLinden *et al*., 2015). Together, these studies provide evidence that *At*NDT1, like *At*NDT2, catalyzes the import of NAD^+^ into the mitochondria under *in vivo* conditions.

Analysis of *A*tndt1‐ and *At*ndt2‐promoter‐GUS plants showed that both genes are strongly expressed in developing tissues, mainly in highly metabolically active cells, which is in line with their mitochondrial localization. However, these transporters have only been characterised at the biochemical and molecular level, while their physiological function remains unclear. For this reason, we aimed to investigate the impact of decreased *AtNDT1* expression on plant development and performance. Given the importance of NAD^+^ in plant metabolism, we would expect that the deficiency of *At*NDT1 transport would impact the redox balance of the cells in different plant tissues. This impact on the redox balance could influence plant growth and development under both optimal and stress situations. In addition, evaluation of the *At*NDT1 protein function is critical for a better understanding of mitochondrial processes and their regulation. To test this hypothesis, we analysed the corresponding Arabidopsis mutants showing reduced expression of *AtNDT1* to understand how and at what level changes in the mitochondrial NAD^+^ transport can affect cell metabolism and plant development. We focused our attention on the impact of reduced *AtNDT1* expression in reproductive tissues and on the importance of this carrier in illuminated leaves.

## Results

### *AtNDT1* is highly expressed in the pollen grain

The need for NAD^+^ for multiple essential functions in cellular organelles is met by specialized NAD^+^ transport proteins. We therefore expect the presence of these carrier proteins in various tissues and at several developmental stages in Arabidopsis. To investigate the *AtNDT1* gene expression, we determined their transcript levels in different organs of young and mature Arabidopsis plants by quantitative real‐time PCR (qRT‐PCR) analysis (Supporting Information Figure S1). In agreement with information in publicly available gene expression databases (http://bar.utoronto.ca), but slightly in contrast to that observed in our previous promotor GUS study (Palmieri *et al*., 2009), *AtNDT1* was strongly expressed in the pollen grain. It also displayed considerable expression in seeds, seedlings, mature leaves and flowers. This suggest that *AtNDT1* transcript is ubiquitously expressed in Arabidopsis, but mainly found in pollen grain. This implies a role in sink and source tissues at any phase of the plant life cycle.

### Generation of Arabidopsis plants with reduced expression of *AtNDT1*

**Figure 1:**
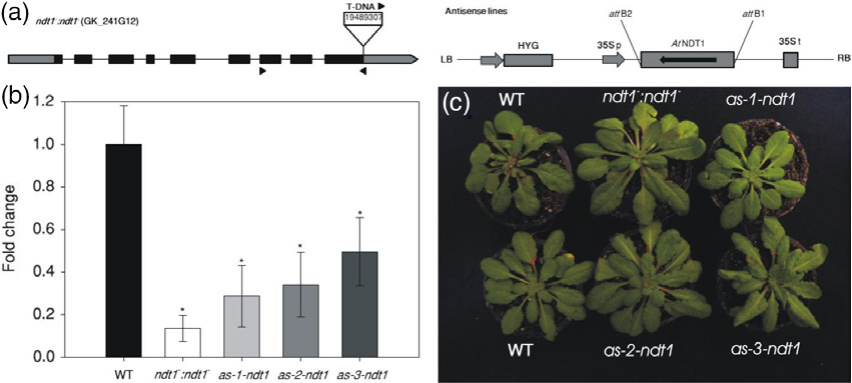
Isolation and characterization of *Arabidopsis thaliana* genotypes deficient in the expression of the mitochondrial NAD^+^ transporter (NDT1). (a) Schematic representation of the gene *AtNDT1* (*At*2g47490) showing the T‐DNA insertion site. The T‐DNA insert, approximately 4.5 kb, is not to scale. Boxes represent gene exons, and arrows on T‐DNA denote primer positions used for population screening. The antisense construct includes the hygromycin resistance gene (*HYG*), the 35S promoter, the gene *NDT1* in antisense position and the 35S terminator. (b) Expression by quantitative real‐time PCR analysis of NDT1 in mature leaves of the *A. thaliana* mutants and wild‐type plants (WT). The values were calculated relative to the WT in rosette leaves of 28‐day‐old plants. Values are presented as mean ± SE of six individual plants per line; an asterisk indicates values that were determined by Student's *t*‐test to be significantly different (*P* < 0.05) from the WT. (c) Phenotypic characterization of 4‐week‐old short‐day grown Arabidopsis genotypes deficient in the expression of the plastidic NAD^+^ transporter (NDT1) and WT plants.

To investigate the physiological role of the *At*NDT1 transporter in Arabidopsis, a T‐DNA insertion line (GK‐241G12) was isolated and three independent antisense lines were selected in the Col‐0 ecotype (Figure 1a). The T‐DNA was inserted in the ninth exon of the *AtNDT1* gene. Due to the presence of the T‐DNA insertion, the homozygous T‐DNA insertion plant *ndt1* ^*−*^ *:ndt1* ^*−*^ contained only 13% of the wild type (WT) *AtNDT1* transcript level in leaves (Figure 1b). The *At*NDT1 antisense lines were generated by ectopic repression of the *AtNDT1* transcription. The *AtNDT1* mRNA levels of the three antisense lines *as‐1‐ndt1*,* as‐2‐ndt1* and *as‐3‐ndt1* were reduced to 28, 34, and 49%, respectively, compared with those in WT leaves (Figure 1b). These independent mutant lines were used for further analyses to investigate the *in planta* function of *At*NDT1.

### Plants with reduced expression of *AtNDT1* display alterations in the expression of genes involved in NAD^+^ metabolism

To provide further insight into the physiological role of *AtNDT1*, we analysed the expression levels of *AtNDT1*,* AtNDT2*, and *AtPXN* in WT and *ndt1* ^*−*^ *:ndt1* ^*−*^ plants by qRT‐PCR using RNA isolated from dry seeds, leaves, flowers and pollen grains. The analysis revealed a significant reduction in the *AtNDT2* transcripts in flowers and pollen grains coupled with increases in the seeds of the *ndt1* ^*−*^ *:ndt1* ^*−*^ mutant compared with their WT counterparts (Figure S2). Moreover, a strong reduction in the expression of *AtPXN* was observed in the *ndt1* ^*−*^ *:ndt1* ^*−*^ pollen grains (Figure S2), while *AtPXN* expression levels were not significantly altered in seeds, leaves and flowers. These data therefore indicated that a reduced *AtNDT1* expression in the *ndt1* ^*−*^ *:ndt1* ^*−*^ is associated with changes in the expression levels of *AtNDT2* and *AtPXN*.

To further characterize the plants with reduced *AtNDT1* expression, the expression of genes encoding NAD^+^ biosynthetic enzymes and NAD kinases was evaluated by qRT‐PCR using RNA isolated from seeds after 48 h of imbibition and leaves from 28‐day‐old rosette (Figure S3). The expression of genes encoding the NAD^+^ biosynthetic enzymes QPRT (quinolinate phosphoribosyltransferase) and NMNAT (nicotinate/nicotinamide mononucleotide adenyltransferase), as well as the genes encoding cytosolic (NADK1) and plastidic (NADK2) isoforms of NAD kinases in Arabidopsis was significantly higher in *ndt1* ^*−*^ *:ndt1* ^*−*^ seeds. These results suggested that, especially in imbibed seeds, the expression of genes related to the biosynthetic pathway of NAD^+^ is upregulated presumably in an attempt to compensate for the lower *AtNDT1* expression. Conversely, expression of the genes encoding enzymes from NAD^+^ metabolism was only slightly lower in *ndt1* ^*−*^ *:ndt1* ^*−*^ mutant leaves (significantly only for the plastidic quinolinate synthase, QS) (Figure S3), which might have occurred as a consequence of different energetic requirements exhibited by seeds and leaves.

### Effects of reduction in *AtNDT1* expression on seed development and seed filling

**Figure 2:**
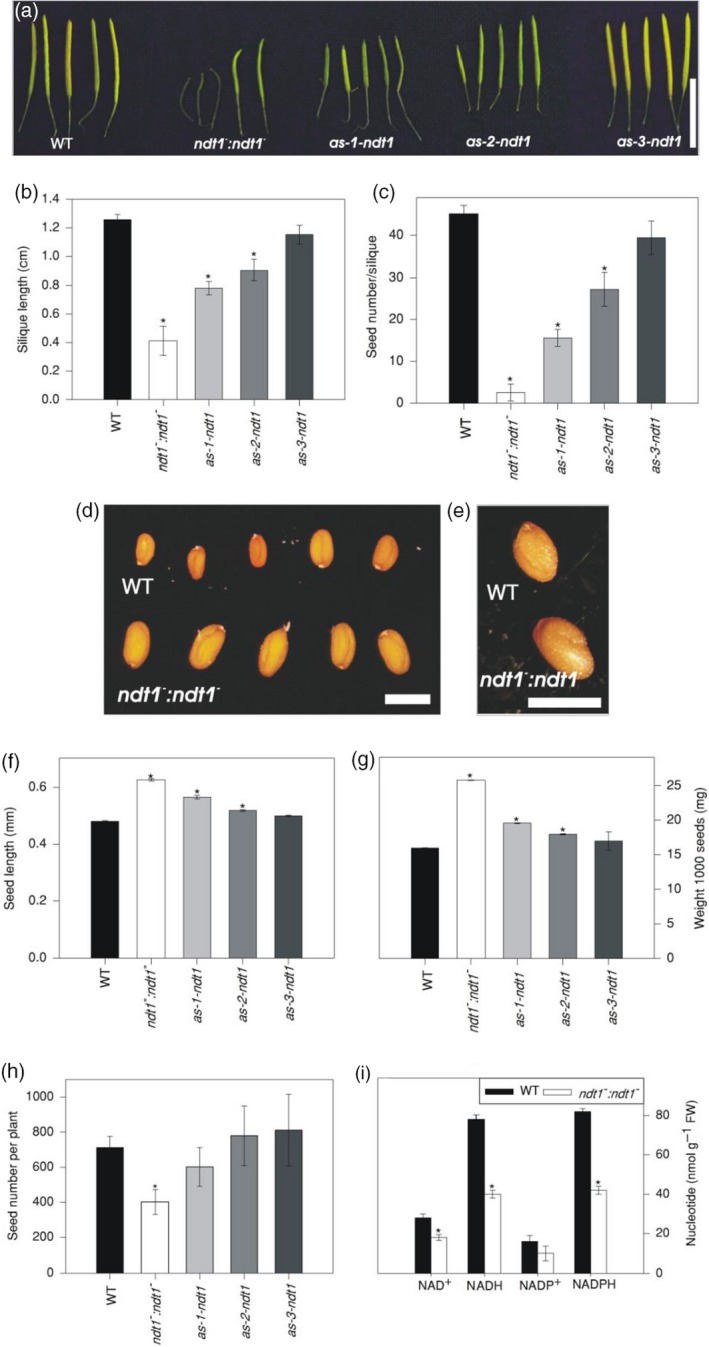
Phenotypic analysis of *Arabidopsis thaliana* genotypes deficient in the expression of the mitochondrial NAD^+^ transporter (NDT1) and wild type (WT) plants. (a) Siliques of all lines. (b) Silique length. (c) Seed number per silique. (d) Seeds of WT and *ndt1^−^:ndt1^−^* showing length differences. (e) Detail of WT and *ndt1^−^:ndt1^−^* seeds. (f) Seed length. (g) Weight 1000 seeds. (h) Seed number per plant. (i) Nucleotide levels in mature siliques. Values are presented as mean ± SE of six individual plants per line; an asterisk indicates values that were determined by *t‐*test to be significantly different (*P* < 0.05) from the WT. FW: fresh weight. Bar in (a) represents 1 cm; and in (d, e), and represent 1 mm.

As *AtNDT1* is expressed in seeds and seedlings, we investigated whether *At*NDT1 plays a role in seed development or seed filling. We observed that the silique size (Figures 2a,b and S4a) and the number of seeds per silique were lower for *ndt1* ^*−*^ *:ndt1* ^*−*^, *as‐1‐ndt1* and *as‐2‐ndt1* lines (Figure 2c). Similarly, the total number of seeds per plant was also decreased in the mutant *ndt1* ^*−*^ *:ndt1* ^*−*^ line (Figure 2h), while seed size increased in these lines compared with WT (Figures 2d–f, and S4b). Moreover, the weight of one thousand seeds from these plants was also higher (Figure 2g), although the total seed weight per plant did not differ among the lines (Figure S4c).

To analyse the accumulation of storage compounds, we also determined lipid, carbohydrate, and protein contents in seeds. *ndt1* ^*−*^ *:ndt1* ^*−*^ mutant line displayed higher amounts of lipids in mature seeds (Figure S5a) and, after imbibition, significantly at 4 days post‐imbibition (Figure S5b). The starch content was also higher in *ndt1* ^*−*^ *:ndt1* ^*−*^ mature seeds (Figure S5c), while similar amounts of proteins (Figure S5d) was observed when compared with WT seeds.

Given the importance of NAD(P)H as a reducing power source for fatty acid biosynthesis, we further analysed how the reduction in the *AtNDT1* expression impacts fatty acid profile in mature seeds (Figure S6). We detected a 2.7‐fold increase of the 14:0 fatty acid in the mutant line compared with WT (Figure S6a), suggesting that NAD^+^ import by *At*NDT1 is required for the metabolism of fatty acids in seeds.

### Effects of reduction in *AtNDT1* expression on seed germination and seedling establishment

To investigate if the increased levels on the storage reserves in the *ndt1* ^*−*^ *:ndt1* ^*−*^ mature seeds have an effect on seed germination and seedling establishment, we evaluated the percentage of seed germination and number of abnormal seedlings. These analyses revealed that germination as well as the percentage of normal developing seedlings were reduced in the *ndt1* ^*−*^ *:ndt1* ^*−*^ line in comparison with WT (Figure S5g,h). In addition, the germination speed index (GSI) and emergence speed index (ESI) were significantly reduced in the *ndt1* ^*−*^ *:ndt1* ^*−*^ line (Figure S5e,f), indicating that *AtNDT1* is an important component affecting seed germination and seedling development.

As NAD^+^ is necessary for the conversion of fatty acids into carbohydrates during storage lipid mobilization to drive seedling establishment (Bernhardt *et al*., 2012), we further evaluated the fatty acids profile in seedlings at 2, 4, and 6 days after imbibition. The TAG marker fatty acid in Arabidopsis is eicosenoic acid C20:1. Interestingly, we observed elevated C20:1 levels in 2‐day‐old, 4‐day‐old and 6‐day‐old mutant seedlings compared with WT, indicating that the repression of *AtNDT1* led to an impaired storage oil mobilization during seedling establishment. Our data also demonstrated that the mutant is able to degrade C20:1, but the degradation rate is slowed down. The amounts of 20:2, 24:0, and 24:1 fatty acids were increased in 2‐day‐old *ndt1* ^*−*^ *:ndt1* ^*−*^ seedlings (Figure S6b). At 4 days after stratification, the fatty acids 14:0 and 20:1a were higher in the mutant seedlings (Figure S6c). In 6‐day‐old seedlings, higher levels of 14:0, 16:2, 16:3, 18:2, 18:3, 20:2, and 20:1a were observed for *ndt1* ^*−*^ *:ndt1* ^*−*^ line compared with WT (Figure S6d). These results suggested that the mobilization of the storage reserves for seed germination rate and seedling establishment is impaired in the mutant plants.

### Effects of reduction in *AtNDT1* expression in pollen

**Figure 3:**
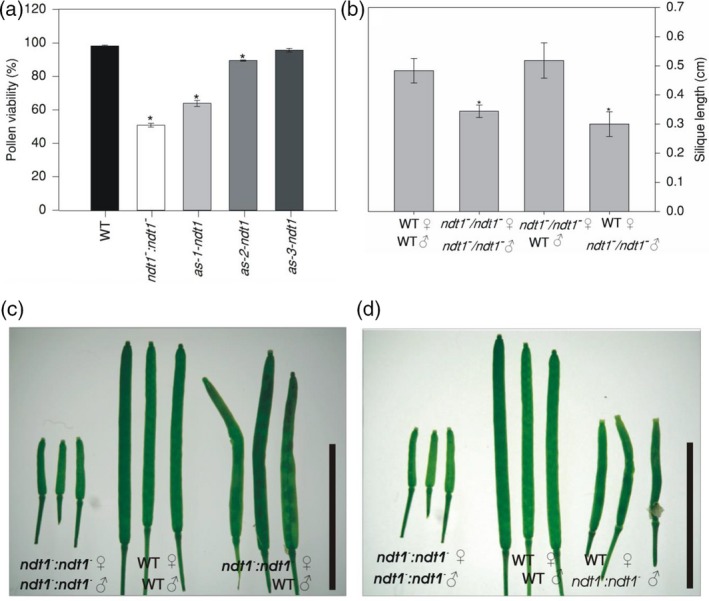
Pollen viability and crossing mutants analyses for phenotypic reestablishment of *Arabidopsis thaliana* genotypes deficient in the expression of the mitochondrial NAD^+^ transporter (NDT1) and wild type (WT) plants. (a) Pollen viability. (b) Silique length in different crossings. (c) Crossing with *ndt1^−^:ndt1^−^* female donor and WT pollen grain donor and controls. (d) Crossing with WT female donor and *ndt1^−^:ndt1^−^* pollen grain donor and controls. Eight crossings were done for each combination and asterisk indicates values that were determined by Student's *t‐*test to be significantly different (*P *< 0.05) comparing WT female donor and WT pollen grain donor with the others crossing. Bars represents 1 cm.

Considering the observed reduction in seed number per silique and the higher expression of *AtNDT1* in the pollen, we evaluated if this phenotype could be a consequence of lower pollen viability, pollen tube growth, effects of lower expression of *AtNDT1* on maternal tissues or impaired embryo development. Comparing WT, *ndt1* ^*−*^ *:ndt1* ^*−*^, *as‐1‐ndt1*, and *as‐2‐ndt1* pollen grains by stereomicroscopy revealed lower pollen grain viability in plants with reduced *AtNDT1* expression reaching only 50, 64, and 89% of the WT values for the *ndt1* ^*−*^ *: ndt1* ^*−*^, *as‐1‐ndt1*, and *as‐2‐ndt1* lines, respectively (Figures 3a and S7). Further analysis revealed that pollen germination and tube growth were also affected in the *ndt1* ^*−*^ *: ndt1* ^*−*^ line (Figure S8). In agreement with these results, *in silico* analysis of the *AtNDT1* gene expression pattern by using the Arabidopsis eFP Brower (Winter *et al*., 2007; http://bar.utoronto.ca/efp/cgi-bin/efpWeb.cgi), indicated that *AtNDT1* is highly expressed in the later stages of pollen development and following germination (Figure S9a,b).

To better understand the reasons underlying the *ndt1* ^*−*^ *:ndt1* ^*−*^ seed phenotype, we pollinated *ndt1* ^*−*^ *:ndt1* ^*−*^ mutants using WT pollen (Figure 3b). Manual pollination of WT (female donor) with *ndt1* ^*−*^ *:ndt1* ^*−*^ pollen (male donor) gave rise to shorter siliques (Figure 3d), while siliques developing on plants in which WT pollen served as male donor used to pollinate *ndt1* ^*−*^ *:ndt1* ^*−*^ (female donor) had no discernible difference in length compared with WT plants (Figure 3c). Therefore, the complementation of *ndt1* ^*−*^ *:ndt1* ^*−*^ stigmas with WT pollen suggested that male gametophyte and pollen development are sensitive to the reduced *At*NDT1 transport activity. Collectively, our results supported the important contribution of *At*NDT1 to pollen viability.

### *AtNDT1* repression enhanced growth and photosynthesis in Arabidopsis plants

The general impact of the reduced expression of *AtNDT1* on vegetative plant growth was studied in detail regarding leaf morphology and photosynthesis. *AtNDT1* T‐DNA insertion and antisense lines were grown under short‐day conditions side by side with WT plants. No differences in the visible phenotype could be identified in 4‐week‐old plants with reduced *AtNDT1* expression (Figure 1c). However, a detailed analysis of the growth parameters revealed significant differences between the transgenic lines when compared with WT (Table 1). The *ndt1* ^*−*^ *:ndt1* ^*−*^ and *as‐1‐ndt1*, the antisense lines with the strongest reduction in *AtNDT1* expression, displayed a higher leaf number (LN) along with increases in the total leaf area (TLA), specific leaf area (SLA), rosette leaf area (RLA), and root system dry weight (RDW). For *ndt1* ^*−*^ *:ndt1* ^*−*^, the specific rosette area (SRA) was also significantly increased compared with WT. For the two other genotypes, representing a lesser reduction in *AtNDT1* transcript, only LN (*as‐2‐ndt1*,* as‐3‐ndt1*) and SLA (*as‐2‐ndt1*) were increased compared with WT.

**Table 1:** Growth parameters of 4‐week‐old Arabidopsis genotypes deficient in the expression of the mitochondrial NAD^+^ transporter (NDT1) and wild type (WT) plants

Parameter	WT	*ndt1* ^*−*^ *:ndt1* ^*−*^	*as‐1‐ndt1*	*as‐2‐ndt1*	*as‐3‐ndt1*
RDW	50.0 ± 3.0	**90.0 **±** **3.0	**80.0 ± 3.0**	60.0 ± 8.0	60.0 ± 4.0
RSDW	10.0 ± 3.0	20.0 ± 3.0	20.0 ± 4.0	10.0 ± 4.0	10.0 ± 3.0
RRS	0.17 ± 0.02	0.18 ± 0.02	0.20 ± 0.01	0.17 ± 0.01	0.19 ± 0.01
LN	13.7 ± 1.4	**19.2 ± 1.5**	**23.0 ± 0.3**	**21.0 ± 1.5**	**19.8 ± 1.3**
TLA	14.64 ± 1.4	**27.27 ± 1.5**	**20.42 ± 0.8**	19.95 ± 2.2	18.65 ± 1.3
SLA	241 ± 24	**320 ± 14**	**306 ± 9**	**331 ± 17**	269 ± 15
RLA	12.8 ± 1.6	**25.7 ± 1.2**	**19.5 ± 0.6**	16.7 ± 1.9	16.8 ± 0.6
SRA	225 ± 51	**303 ± 11**	266 ± 5	279 ± 25	252 ± 12
Stomatal density	138.4 ± 4.8	**117.9 ± 3.3**	**117.9 ± 2.4**	121.9 ± 5.2	136.6 ± 10.1

RDW, rosette dry weight (mg); RSDW, root system dry weight (mg); RRS, root/shoot ratio; LN, leaf number; TLA, total leaf area (cm^2^); SLA, specific leaf area (cm^2^ g^−1^); RLA, rosette leaf area (cm^2^); SRA, specific rosette area (cm^2^ g^−1^) and stomata density (stomatal number mm^−2^).

Values are presented as means ± SE of determinations on six individual plants per line; bold type values were determined using Student's *t*‐test to be significantly different (*P *<* *0.05) from the WT.

**Figure 4:**
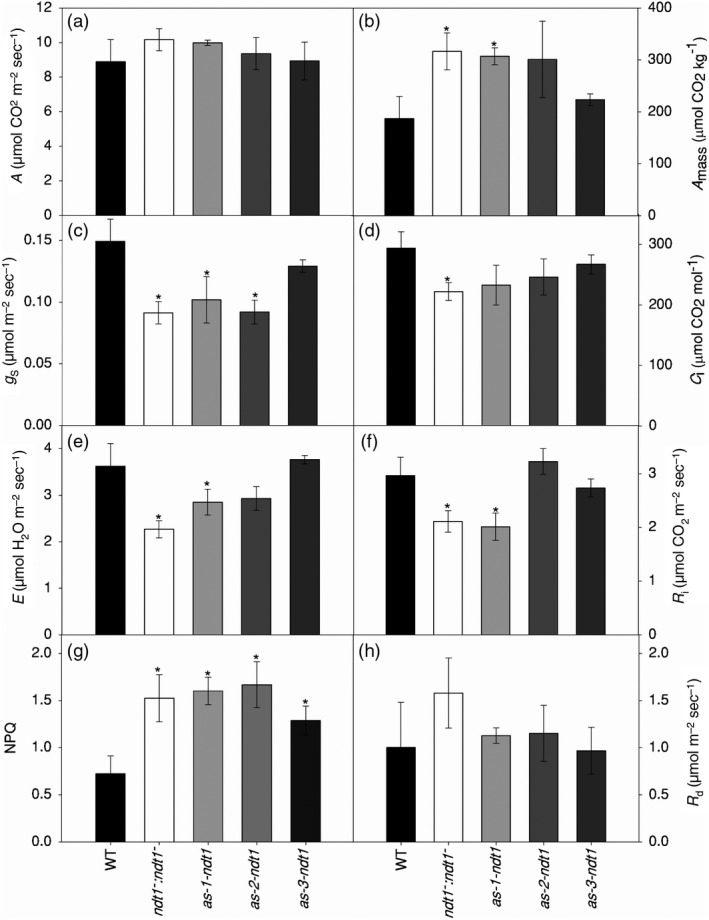
Gas exchange and chlorophyll *a* fluorescence parameters in 4‐week‐old *Arabidopsis thaliana* genotypes deficient in the expression of the mitochondrial NAD^+^ transporter (NDT1) and wild type (WT) plants. (a) Assimilation rate (*A*) per area unit at 400 μmol photons m^2^ s^−1^. (b) Assimilation rate (*A*_mass_) per mass unit at 400 μmol photons m^−2^ sec^−1^. (c) Stomatal conductance (*g*_s_). (d) Internal CO_2_ concentration (*C*_i_). (e) Transpiration (*E*). (f) Photorespiration (*R*_i_). (g) Nonphotochemical quenching (NPQ). (h) Dark respiration (*R*_d_). Values are presented as mean ± SE of six individual plants per line; an asterisk indicates values that were determined by *t‐*test to be significantly different (*P* < 0.05) from the WT.

To investigate if the repression of *AtNDT1* affects photosynthesis, several photosynthetic parameters were measured in the mutant plants. A reduced *AtNDT1* expression did not alter CO_2_ assimilation rates at 400 μmol photons m^−2^ sec^−1^ (Figure 4a), or at 100 μmol photons m^−2^ sec^−1^ (Figure S10a,b). The parameters obtained from the light response curves (Figure S10a) were similar to WT when calculated as per unit of leaf area (Table S1). We further normalised the photosynthetic rates to be expressed per unit of mass, taking the leaf mass and thickness into account, sas increased SLA was observed in the mutant lines (Table 1). Interestingly, photosynthetic rates increased significantly in the *ndt1* ^*−*^ *:ndt1* ^*−*^ and *as‐1‐ndt1* plants (Figure 4b), whereas stomatal conductance (*g*_s_), internal CO_2_ concentration (*C*_i_), transpiration (*E*), photorespiration (*R*_*i*_), and stomatal density decreased in the NDT1 deficient plants (Figure 4c–f). Furthermore, non‐photochemical quenching (NPQ) increased in *ndt1* ^*−*^ *:ndt1* ^*−*^, *as‐1‐ndt1*,* as‐2‐ndt1* and *as‐3‐ndt1* plants relative to WT plants under 400 μmol photons m^−2^ sec^−1^ and higher light intensities (Figure 4g; Figure S11). In contrast, the electron transport rate (ETR), maximum quantum efficiency of photosystem II (*F*_v_/*F*_m_), the instantaneous water use efficiency (*A*/*E*), and intrinsic water use efficiency (*A*/*g*_s_) did not change significantly compared with WT (Figure S10c–f). These results indicated that the enhanced growth of the *ndt1* mutant plants is linked with an increased photosynthetic activity.

### Reduction of *AtNDT1* expression affected starch and sugar accumulation and altered cellular redox poise in leaves

**Figure 5:**
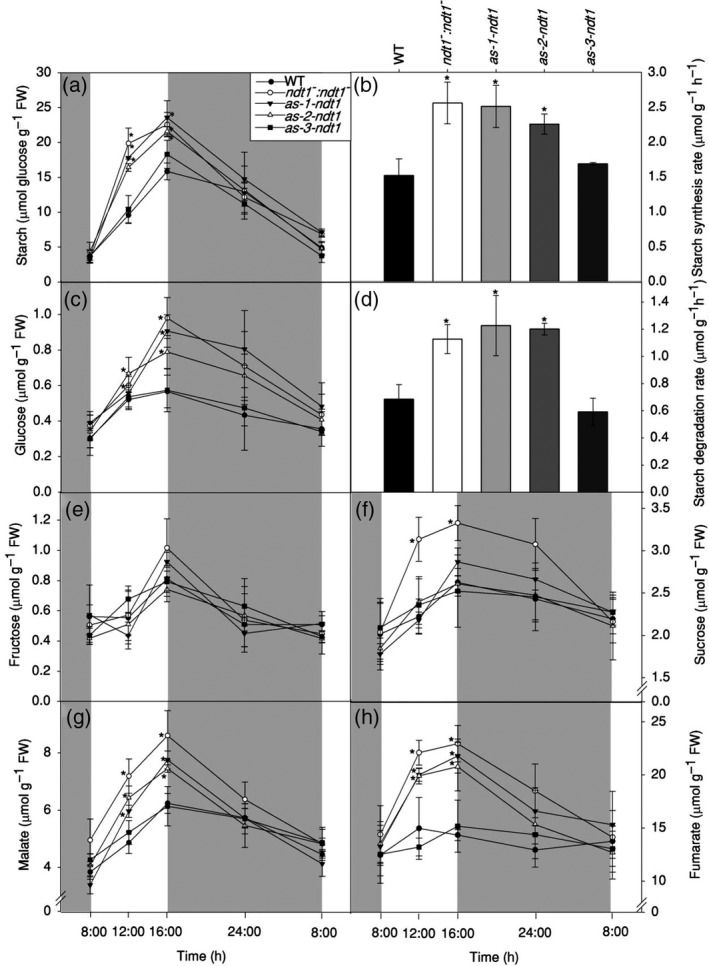
Leaf metabolite levels of 4‐week‐old *Arabidopsis thaliana* genotypes deficient in the expression of the mitochondrial NAD^+^ transporter (NDT1) and wild type (WT) plants. (a) Starch. (b) Starch synthesis rate. (c) Glucose. (d) Starch degradation rate. (e) Fructose. (f) Sucrose. (g) Malate. (h) Fumarate. Values are presented as mean ± SE of six individual plants per line; an asterisk indicates values that were determined by *t‐test* to be significantly different (*P* < 0.05) from the WT. Grey areas represent the dark period. FW: fresh weight.

To evaluate the putative metabolic changes caused by *AtNDT1* silencing in fully expanded leaves, compounds related to carbon and nitrogen metabolism were measured throughout the diurnal cycle. Plants with reduced *AtNDT1* transcript levels accumulated more starch at the end of the light period (Figure 5a). Accordingly, increased starch synthesis and starch degradation rates were observed for the mutant plants (Figure 5b,d). Glucose and sucrose levels were higher in the *ndt1* ^*−*^ *:ndt1* ^*−*^ mutant plants in the middle and in the end of the light period (Figure 5c,f), while fructose levels remained unaltered (Figure 5e). Interestingly, malate and fumarate contents also increased throughout the day in the *ndt1* ^*−*^ *:ndt1* ^*−*^, *as‐1‐ndt1*, and *as‐2‐ndt1* plants (Figure 5g,h). Protein content was increased in the *ndt1* ^*−*^ *:ndt1* ^*−*^ line at the end of the light period (Figure S12), whereas plants with low *AtNDT1* expression levels did not display altered chlorophyll levels (Figure S13).

A non‐targeted metabolic profile analysis identified additional effects in leaf samples harvested in the middle of the light period (Table S2). Among the successfully annotated metabolites, sugars and related compounds differed markedly in the lines with low *AtNDT1* expression. *AtNDT1* silencing led to elevated levels of glucose (significantly in all lines), fructose (significantly in *ndt1* ^*−*^ *:ndt1* ^*−*^ and *as‐3‐ndt1* lines), and galactinol (significantly in *ndt1* ^*−*^ *:ndt1* ^*−*^, *as‐2‐ndt1* and *as‐3‐ndt1* lines). Additionally, glutamate (significantly in *ndt1* ^*−*^ *:ndt1* ^*−*^ and *as‐1‐ndt1* lines), leucine (significantly in *ndt1* ^*−*^ *:ndt1* ^*−*^ line), sorbose (significantly in *ndt1* ^*−*^ *:ndt1* ^*−*^ line), erythritrol (significantly in *ndt1* ^*−*^ *:ndt1* ^*−*^ line), and myo‐inositol (significantly in *ndt1* ^*−*^ *:ndt1* ^*−*^ and *as‐3‐ndt1* lines) levels were elevated in the *AtNDT1* silencing lines. Furthermore, the levels of ascorbate were also increased in leaves of *ndt1* ^*−*^ *:ndt1* ^*−*^ and *as‐1‐ndt1* lines.

**Figure 6:**
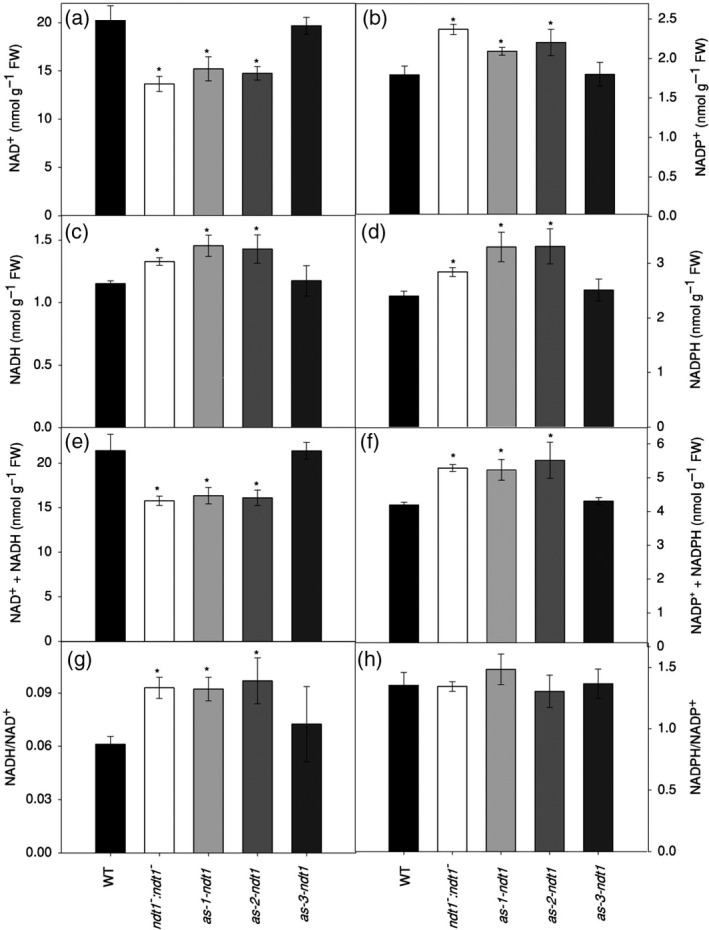
Changes in nucleotide levels in fully expanded leaves of 4‐week‐old *Arabidopsis thaliana* genotypes deficient in the expression of the mitochondrial NAD^+^ transporter (NDT1) and wild type (WT) plants, collected at midday. (a) NAD^+^. (b) NADP^+^. (c) NADPH. (d) NADPH. (e) NADH/NAD^+^ ratio. (f) NADPH/NADP^+^ ratio. Values are presented as mean ± SE of six individual plants per line; an asterisk indicates values that were determined by *t*‐test to be significantly different (*P* < 0.05) from the WT. FW: fresh weight.

An analysis of the pyrimidine nucleotide pools in leaves (Figure 6) revealed a significant decrease in the contents of NAD^+^ in *ndt1* ^*−*^ *:ndt1* ^*−*^, *as‐1‐ndt1*, and *as‐2‐ndt1* lines (Figure 6a), while the levels of NADP^+^, NADH and NADPH increased in the same lines compared with WT (Figure 6b–d). Furthermore, the sum of NAD^+^ and NADH decreased, while the NADP^+^ plus NADPH increased significantly in *ndt1* ^*−*^ *:ndt1* ^*−*^, *as‐1‐ndt1*, and *as‐2‐ndt1* (Figure 6e–f). Interestingly, a higher NADH/NAD^+^ ratio was observed in these lines compared with WT (Figure 6g), while the NADPH/NADP^+^ ratio was not significantly altered (Figure 6h). To confirm changes in the cellular redox state in the mutant plants, we evaluated the activation state of NADP‐dependent malate dehydrogenase (NADP‐MDH) (Table 2), a key redox‐regulated enzyme controlling the malate valve, which exports reducing equivalents indirectly from chloroplasts. We observed an increase in the NADP‐MDH activation state in mutant leaves, which was significantly different for *ndt1* ^*−*^ *:ndt1* ^*−*^ and *as‐1‐ndt1*. The significant increase in the maximal NADP‐MDH activity corroborated well with the increased NADPH (Figure 6d) and malate levels (Figure 5g) found in plants with reduced expression of *AtNDT1*. Altogether, these results suggested that the cellular redox state is changed as a consequence of the reduced expression of *AtNDT1*.

**Table 2:** NADP‐dependent malate dehydrogenase (NADP‐MDH) of 4‐week‐old Arabidopsis mutants deficient in the expression of the mitochondrial NAD^+^ transporter (NDT1) and wild type (WT) plants

Enzymes	WT	*ndt1* ^*−*^ *:ndt1* ^*−*^	*as‐1‐ndt1*	*as‐2‐ndt1*	*as‐3‐ndt1*
NADP‐MDH initial^a^	12.7 ± 0.2	**13.5 ± 0.2**	12.8 ± 0.7	13.2 ± 0.5	12.8 ± 0.4
NADP‐MDH total^a^	16.0 ± 0.5	14.8 ± 0.3	14.3 ± 0.7	15.6 ± 1.0	14.7 ± 0.9
NADP‐MDH activation state^b^	79.7 ± 2.0	**91.2 ± 1.4**	**89.6 ± 3.6**	85.2 ± 3.2	87.6 ± 2.9

Activities were determined in whole rosettes harvested at the middle of the photoperiod. Values are presented as mean ± SE (*n* = 6); values in bold type in mutant plants were determined by using Student's *t*‐test to be significantly different (*P *<* *0.05) from the WT.

FW, fresh weight.

a μmol min^−1^ g^−1^ FW.

b Percentage of NADP‐MDH total.

### Subcellular localization of *At*NDT1‐GFP revealed its mitochondrial localization

**Figure 7:**
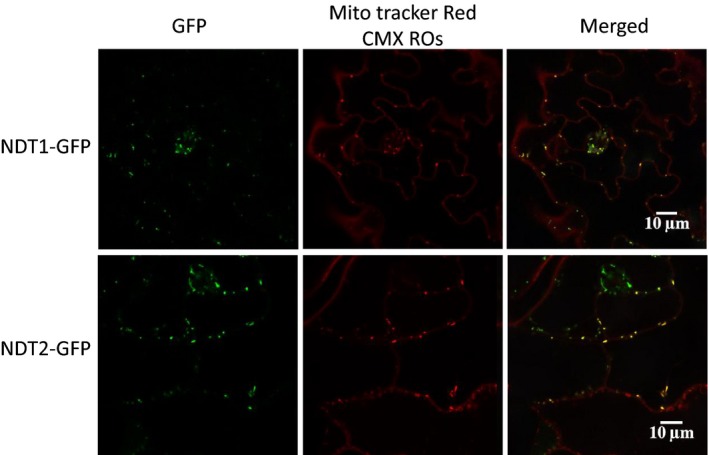
Localization of *At*NDT1 and *At*NDT2 by confocal laser scanning microscopy (CLSM). Arabidopsis plants were stable transformed with C‐terminal GFP fusions of NDT1 (upper panel) and NDT2 (lower panel), respectively. Whole seedlings were analyzed by CLSM. The left panel shows the GFP‐specific fluorescence signal (green) while the middle panel shows the localization of the mitochondrial Mito tracker Red CMX ROs (red). The right panel represents the merged image of both channels revealing an overlay of the fluorescent signals (yellow) indicating a mitochondrial localization of NDT1 and NDT2.

*At*NDT1 was presumed to exclusively reside in the chloroplast membrane (Palmieri *et al*., 2009). However, this protein was recently found in mitochondrial membranes in proteome studies (Senkler *et al*., 2017) and a previous GFP‐tagging and immunolocalization study was not able to find *At*NDT1 targeted to chloroplast membranes (Bedhomme *et al*., 2005). In addition, many phenotypic changes (described above) caused by *AtNDT1* downregulation did not directly support that *At*NDT1 functions in the chloroplast but rather suggested an increase in chloroplast import of NAD^+^ at the expense of deficient mitochondria import. Considering these facts, we decided to re‐evaluate the subcellular localization of *At*NDT1. To provide experimental evidence on the subcellular localization of *At*NDT1 and *At*NDT2, we generated corresponding GFP fusions under the control of the ubiquitin 10 promotor (Grefen *et al*., 2010) and expressed the recombinant proteins in Arabidopsis seedlings. Both *At*NDT1‐ and *At*NDT2−GFP fusion proteins were exclusively localized in the mitochondria (Figure 7), indicating their mitochondrial localization.

## Discussion

### *At*NDT1 has a mitochondrial localization in Arabidopsis

It has been previously described that *At*NDT1 protein resides in the chloroplast membrane (Palmieri *et al*., 2009). Intriguingly, however, both *At*NDT1 and *At*NDT2 have been found in mitochondria by a recent proteome study (Senkler *et al*., 2017) and could not be localized to plastids by earlier GFP‐tagging and/or immunolocalization studies (Bedhomme *et al*., 2005). Moreover, our results revealed unexpected physiological features exhibited by *AtNDT1* downregulated lines that are not consistent with the proposed chloroplastic localization of *At*NDT1. For instance, the higher photosynthetic rates (Figure 4), the increased NADP^+^ and NADPH contents in leaves (Figure 6), and the higher accumulation of sugar and starch at the end of the light period in the mutants (Figure 5) in comparison with their WT counterparts implicated an increase in the chloroplast import of NAD^+^. The observed increase in the activation state of the plastidic NADP‐MDH (Table 2) also contradicted the proposed chloroplastidic localization of *At*NDT1.

Given that the information on correct subcellular localization is essential to deduce the effect of a protein on metabolism (Kirchberger *et al*., 2008), we re‐investigated the question in which membrane *At*NDT1 resides. Considering the similarities in the biochemical properties of *At*NDT1 and *At*NDT2, Arabidopsis plants were stably transformed with C‐terminal GFP fusions of either *At*NDT1 or *At*NDT2. Both *At*NDT1‐ and *At*NDT2‐GFP fusion proteins were found to exclusively localize in the mitochondria (Figure 7). Considering the discrepancy with some of the earlier work on this protein, we can only speculate the possible reasons why previous studies (Palmieri *et al*., 2009) failed to detect NDT1 proteins in the mitochondrial compartment. The fact that the previously published chloroplast membrane location of *At*NDT1 was deduced from interpretation of the location of an *At*NDT1‐GFP fusion protein transiently expressed in tobacco leaf protoplasts (Palmieri *et al*., 2009) and not in Arabidopsis plants may possibly explain why previous investigations failed to detect the mitochondrial localization of *At*NDT1–GFP proteins. Based on these findings, we proposed to re‐evaluate the physiological roles of *At*NDT1 within plant cells and its connection to metabolic and redox‐mediated control of cellular processes in light of its newly found mitochondrial localization.

### *At*NDT1 is involved in the cellular NAD^+^ homeostasis

The fact that the pyridine pools are to some extent interdependent (Agledal *et al*., 2010) raises the question of the very large range of possible consequences of the decreased NAD^+^ import into mitochondria. Therefore, to investigate the role of the mitochondrial NAD^+^ carrier *At*NDT1, we analyzed the phenotypes of corresponding Arabidopsis mutant and antisense plants. Here, we show that plants with lower expression of the *AtNDT1* displayed reductions in the NAD^+^ levels in leaves (Figure 6a) and in siliques (Figure 2i), whereas the levels of NADH, NADP^+^ and NADPH were increased (Figure 6b–d, respectively). Furthermore, the ratio NADH/NAD^+^ was shown to be augmented in plants that carried reductions in *At*NDT1 expression, leading these plants to a more reduced state (Figure 6g). That said, the observed reduction in the levels of NAD^+^ may also be associated with changes in the rate of NAD^+^ biosynthesis in the plants with reduced *AtNDT1* expression. In bacteria, regulation of NAD^+^ levels occurs at the level of transcription (Penfound and Foster, 1999). These organisms contain genes belonging to NAD^+^ biosynthesis organized in *operons* and the content of NAD^+^ regulates their expression (Penfound and Foster, 1999). However, little information is known about the regulation of NAD^+^ content at the level of gene expression in plants. Therefore, to test whether altered NAD^+^ content in plants with decreased expression of *AtNDT1* would influence gene expression, we quantified the expression levels of genes that encoded enzymes belonging to NAD^+^ salvage and *de novo* pathways (Figure S3). This analysis revealed that *ndt1* ^*−*^ *:ndt1* ^−^ plants displayed differential expression of genes encoding the NAD^+^ biosynthetic pathway; *QPRT* and *NMNAT* showed higher expression in imbibed seeds, while *QS* expression was reduced in *ndt1* ^*−*^ *:ndt1* ^−^ leaves. Furthermore, higher expression of *AtNDT2* in imbibed seeds and a minor increase in leaves (Figure S2) of *ndt1* ^*−*^ *:ndt1* ^−^ line, was observed. Higher *At*NDT2 expression may be acting as a compensatory mechanism for NAD^+^ import into mitochondria in plants carrying reduced *At*NDT1 expression; which implied that *At*NDT1 and *At*NDT2 may have overlapping functions in the NAD^+^ transport in seeds and leaves. Moreover, these results suggested that NAD^+^ metabolism and transport in the mitochondrion and peroxisome were modified in those plants. Interestingly, NADP^+^ and NADPH levels were increased with decrease in the sum of NAD^+^ and NADH (Figure 4), indicating that NAD^+^ phosphorylation is likely to be promoted by *AtNDT1* downregulation. Given that we found no changes in the expression of the NAD kinases (NADK1‐3) in leaves (Figure S3b), and considering the mild increase of *AtNDT2* expression (which was strongly expressed in *NDT1* mutant seeds), it can be presumed that the compensatory increased expression of the mitochondrial NAD^+^ carrier *AtNDT2* is mainly responsible for the observed rise in the NADH/NAD^+^ ratio in the leaves of *AtNDT1* mutants. Taken together, these results suggested that *At*NDT1 is likely to play an important role in the cellular NAD^+^ homeostasis in plants by linking pyridine nucleotide pools in different subcellular compartments. Therefore, as a consequence of *AtNDT1* downregulation, whole NAD^+^ metabolism and transport could be re‐balanced.

### Reduced *AtNDT1* expression impacts carbon metabolism turnover in the light and photosynthesis

Here, we show that the reduction in *AtNDT1* expression influenced processes that take place in chloroplasts, suggesting that there is a precise communication between organelles regarding NAD^+^ levels in subcompartments. We demonstrated that the downregulation of *AtNDT1* increased the photosynthetic rate per unit of mass (Figure 4b), impacted leaf carbon metabolism (Figure 5), and enhanced plant growth (Table 1). Moreover, NPQ was increased in *ndt1* ^*−*^ *:ndt1* ^*−*^, *as‐1‐ndt1*,* as‐2‐ndt1* and *as‐3‐ndt1* plants under high light (400–1200 μmol photons m^−2^ sec^−1^) (Figure S10), which would imply a decrease in chloroplastic NADP^+^ levels under these conditions (Takahashi *et al*., 2006). This finding is in line with the observed increase in the activation state of the plastidic NADP‐MDH (Table 2), as this enzyme uses excess NADPH to regenerate the electron acceptor NADP^+^ and is inhibited by the product NADP^+^ (Scheibe, 2004). In support of this hypothesis, plants with reduced *AtNDT1* expression exhibited a higher NADH/NAD^+^ ratio (Figure 6a) and increase in NADPH in leaves (Figure 6d).

Redox regulation is the preferred strategy for plastidic enzymes to regulate a range of metabolic processes such as carbon fixation, starch metabolism, lipid synthesis and amino acid synthesis (Geigenberger *et al*., 2005). The time course analysis provided here clearly demonstrated a pronounced accumulation of glucose, starch, fumarate, and malate during the day, but not at night, in leaves of the mutants with reduced *AtNDT1* expression (Figure 5). Taking into account that NADP‐MDH is a redox‐controlled enzyme only active in the light, these results reinforced the hypothesis that the malate valve is being used by the mutants to balance cellular energy supply (Scheibe, 2004). Furthermore, the increase in the activation of NADP‐MDH and in NPQ values indicated that low *AtNDT1* expression led to a energetic reduced state of the chloroplast that could be associated with the increase in starch biosynthesis (Figure 5b) and photosynthesis per mass unit (Figure 4b).

Photosynthetic capacity may be limited either by biochemical or diffusive processes. The first refers to CO_2_ fixation by RuBisCO and the second to stomatal and mesophyll resistances that CO_2_ can encounter during its diffusion from the atmosphere to the carboxylation sites in the chloroplasts (Flexas *et al*., 2008). The reverse of stomatal resistance is *g*_s_ and changes in this parameter may occur in parallel with changes in stomatal density (Tanaka *et al*., 2013). Despite decreases in stomatal density (Table 1) and *g*_s_ (Figure 4c) in the mutant lines, the increases in photosynthesis demonstrated that the mutant plants maintained the synthesis of NADP^+^ required for photochemical reactions that might be explained by the indirect transfer of redox equivalents by redox shuttles from the cytosol across the chloroplast membrane. Curiously, reduced *AtNDT2* expression negatively affected photosynthetic efficiency as a result of a lowered stomatal density and conductance (unpublished work). This apparent contradiction can be explained by the fact that *AtNDT1* expression is much higher than *AtNDT2* in leaves (Figure S2). Therefore, the entire NAD^+^ metabolism and transport seems to be reorganized in *AtNDT1* downregulated lines in a more extended way than that in *AtNDT2* mutant lines, in a manner that enables greater transfer of redox power for photochemical reactions, therefore compensating lower stomatal conductance and density in leaves of *AtNDT1* downregulated lines.

*AtNDT1* was highly expressed in guard cells (Figure S9c); this finding is in line with the lower stomatal conductance observed in *ndt1* ^*−*^ *:ndt1* ^*−*^ plants in our study (Figure 4c). It has been shown that NAD^+^ levels are reduced in response to the generation of ROS induced by abscisic acid (ABA), which acts as a signal for stomatal closure (Hashida *et al*., 2010). Therefore, considering the association of NAD^+^ metabolism to stomatal function, the results presented here suggested that NAD^+^ is important not only for stomatal movements, but that NAD^+^ transport also acts in regulating guard cell biogenesis.

### Repression of *AtNDT1* affects pollen viability

It has been demonstrated that pollen maturation and tube growth are dependent on energy produced by mitochondrial respiration and plastid glycolysis (Selinski and Scheibe, 2014). Recently, it has been shown that NADPH provision via the OPPP in peroxisomes is also needed for gametophytic interaction during pollen tube guidance to ovules (Hölscher *et al*., 2016). Therefore, it is clear that energy metabolism during pollen maturation and tube growth is highly complex and involves different pathways and cell compartments and, as NAD^+^ supply is essential for reduction related reactions, NAD^+^ transport is necessary. For example, deficiency in the plastidic glycolytic glyceraldehyde‐3‐phosphate dehydrogenase, an enzyme that reversibly converts the glyceraldehyde‐3‐phosphate to 1,3‐bisphosphoglycerate, with the reduction of NAD^+^ to NADH, displays male sterility in Arabidopsis, due to a disorganized tapetum cell layer (Muñoz‐Bertomeu *et al*., 2010). Furthermore, NAD(P)H accumulation during the growth of the pollen tube is directly coupled to ATP generation, which is used to enable a variety of energy‐dependent processes localized in the pollen tube tip (Cárdenas *et al*., 2006).

Recent studies using mutants deficient in NAD^+^ biosynthesis demonstrated a functional importance of NAD^+^ in reproductive tissues such as pollen, floral meristem, siliques and seeds (Hashida *et al*., 2007, 2013; Hunt *et al*., 2007; Liu *et al*., 2009). Here, we demonstrated that *AtNDT1* silencing promoted a decrease in pollen viability and pollen tube growth (Figures 3a, S7 and S8). Cumulative evidence supports the importance of NAD^+^ metabolism in pollen grain formation and pollen tube growth (Berrin *et al*., 2005; Chai *et al*., 2005, 2006; Cárdenas *et al*., 2006; Hashida *et al*., 2009). In freshly harvested dry pollen, NAD^+^ is accumulated, whereas it dramatically decreased immediately after water contact (Hashida *et al*., 2013). Furthermore, several metabolic pathways, which operate during maturation of pollen grains and pollen tube growth such as the biosynthesis of lipids, components of cell walls, and amino acids, are highly dependent on NAD^+^ levels (Hashida *et al*., 2009). At the same time, the occurrence of high NAD^+^ levels can maintain dormant pollen grains by competitive inhibition of NADH‐dependent redox reactions that are essential for the formation of the pollen tube (Hashida *et al*., 2013). Accordingly, the formation of non‐viable pollen as found in our study and the strong expression of *AtNDT1* in pollen during the later stages of development and following germination (Figure S9) confirmed that *At*NDT1 is of fundamental importance for pollen formation and germination.

In this study, we have shown that decreased *AtNDT1* expression also resulted in reduced expression of *AtPXN* and *AtNDT2* in pollen grains (Figure S2). This result indicated that reduction in NAD^+^ import by *At*NDT1 may also impact the NAD^+^ import by *At*NDT2 and *At*PXN and this may possibly disturb the whole cell NAD^+^ balance in pollen. It deserves special mention that *AtNDT2* expression is dramatically lower than *AtNDT1* in the pollen of WT Arabidopsis (Figure S2), meaning that emerging phenotypes in pollen could directly result from *AtNDT1* downregulation.

### Seed germination and seedling establishment are impaired due to *AtNDT1* repression

The decreased viability of pollen grains observed in plants with low *AtNDT1* expression probably contributed to the significant reduction in the number of seeds and the resulting reduction in siliques filling and lower length and diameter of the siliques (Figures 2 and S4). The production of shorter siliques can be ascribed to misfunctional male gametophytes (Hashida *et al*., 2013). It was recently found that mutant plants deficient in the expression of nicotinamide mononucleotide adenylyltransferase (NMNAT), an important enzyme in NAD^+^ biosynthesis, produced non‐viable pollen grains, shortened siliques, and smaller numbers of seeds per plant (Hashida *et al*., 2013). These authors did not comment on the mechanism that led to the shortening of siliques, although, consistent with our observations, they showed that it may be a result of pollen germination and malformation of the pollen tube, due to the lower concentration of NAD^+^ found in the mutants.

The lower number of seeds in *ndt1* ^*−*^ *:ndt1* ^*−*^ plants suggested that, in addition to NAD^+^ metabolism, mitochondrial NAD^+^ transport is also important during embryonic development. As the concentration of nucleotides decreased in mature siliques (Figure 2i), we may assume that resources allocated to the zygote were impaired. As a result, fewer seeds were generated, however the seeds produced received a greater amount of carbohydrates, explaining the larger seed size in *ndt1* ^*−*^ *:ndt1* ^*−*^, *as‐1‐ndt1* and *as‐2‐ndt1* plants (Figures 2 and S4b).

Interestingly, the repression of *AtNDT1* expression was associated with a higher amount of lipids per seed (Figure S5a). It is well known that Arabidopsis seeds have high fatty acid content (~35% by weight; Baud *et al*., 2002), which serve as a carbon and energy reserve. Accordingly, the increase in lipid content per seed was followed by a mild but non‐significant increase in starch contents in *ndt1* ^*−*^ *:ndt1* ^*−*^ seeds, without changes in the protein level (Figure S5c,d). Taken together, these results suggested that the remaining seeds apparently exerted a higher sink strength and therefore accumulated a higher amount of storage compounds when the *AtNDT1* expression was reduced. Additionally, the relative concentration of fatty acids alongside seedling development was generally higher in the *ndt1* ^*−*^ *:ndt1* ^*−*^ line (Figure S6). As NAD^+^ is required for β‐oxidation during lipid mobilization and seedling development (Bernhardt *et al*., 2012), it is suggested that NDT1 mutant seeds fail to mobilize lipids possibly as a consequence of an impaired fatty acid β‐oxidation. Therefore, the delay in seed germination and impaired fatty acid degradation (β‐oxidation) may also indicate changes in the peroxisomal NAD^+^ status in the *ndt1* ^*−*^ *:ndt1* ^*−*^ line. Additionally, given that seed dormancy may be regulated by the relative levels of pyridine nucleotides (Hunt and Gray, 2009), it is possible that the reduced germination rates observed (Figure S5) are related to alterations in the levels of NAD(P)(H) in the mutant siliques (Figure 2i).

### *At*NDT1 and *At*NDT2 show varying degrees of redundancy and specialization

The demonstration that both NDT1 and NDT2 are localized in the mitochondria of Arabidopsis raises questions about their exact roles *in vivo*. Therefore, to ascertain the individual contributions of *At*NDT1 and *At*NDT2 to metabolism, it is important to reconsider previously reported biochemical features of the two carriers (Palmieri *et al*., 2009). First, *At*NDT1 and *At*NDT2 genes exhibited high structural similarities and their encoded proteins shared a number of similar transport properties, with both accepting AMP and ADP as highly efficient counter‐exchange substrates for NAD^+^. Second, it is assumed that they derive from a common evolutionary ancestor; this assumption could explain similarities in their biochemical properties (Palmieri *et al*., 2009). Third, in spite of these functional similarities, *At*NDT1 and *At*NDT2 have different kinetic constants, with different levels of activity and affinities for NAD^+^ (Palmieri *et al*., 2009). In addition, it should be recalled that *AtNDT1* and *AtNDT2* display distinct tissue‐specific expression patterns. For instance, gene expression analysis of *AtNDT1* and *AtNDT2* by qRT‐PCR (Figure S2) demonstrated much higher expression of *AtNDT1* than *AtNDT2* in seeds, leaves, flowers and pollen, therefore suggesting that *At*NDT1 is the dominant NDT isoform in *A. thaliana* in these tissues. In addition, gene expression analysis of *AtNDT1* and *AtNDT2* (Figure S2), and depicted by the publicly available data in Arabidopsis eFP Browser, demonstrated that the highest expression of *AtNDT1* occurs in pollen grains in comparison with its expression in seeds, leaves and flowers. Another aspect to be pointed out is that the T‐DNA insertion lines for *AtNDT1* and *AtNDT2* showed reductions in pollen grain viability of approximately 50% and 13%, respectively, in relation to the corresponding WT. Altogether, these data suggested that the formation of non‐viable pollen in *AtNDT1* downregulated lines is mainly a result of *AtNDT1* downregulation. Moreover, given that decreased *AtNDT1* expression also resulted in lower expression of *AtNDT2* in pollen grains (Figure S2), it seems that, at least in pollen, *AtNDT2* does not compensate for *AtNDT1* deficiency. Notwithstanding, the mutant plants for either *AtNDT1* or *AtNDT2* exhibited reductions in pollen viability, seed germination, stomatal density and conductance, indicating that the activities of both *At*NDT1 and *At*NDT2 are important for NAD^+^ metabolism in these tissues (unpublished work and the current study). In spite of these similarities, mutant plants for *AtNDT1* and *AtNDT2* displayed opposite phenotypes regarding their photosynthetic performance, having higher and lower photosynthetic rates, respectively, than the corresponding WT plants. In conjunction, this information demonstrated that, even though *At*NDT1 and *At*NDT2 cooperatively participate in NAD^+^ import into mitochondria, both proteins have varying degrees of specialization which probably took place alongside their independent cellular evolution and that have allowed the development of their individual properties such as the different transport properties and gene expression patterns.

In summary, the present study revealed that *At*NDT1 is targeted to the inner mitochondrial membrane and that this transport protein appears to play an important role in cellular NAD^+^ homeostasis in leaves. As a consequence of *AtNDT1* downregulation, NAD^+^ metabolism and transport seems to be reorganized, leading to metabolic shifts, which results in increases in photosynthesis, starch accumulation and in the activation state of the stromal NADP‐MDH. In addition, impaired *At*NDT1 transport results in reduced pollen grain viability, tube growth, and seed filling, demonstrating the important role of *At*NDT1 in reproductive tissues. Furthermore, we demonstrated a possible function for NAD^+^ transport during seed germination and seedling establishment that appears to be linked to the need of NAD^+^ during lipid metabolism and related processes. In the future, we are interested in identifying to what extent, *At*NDT1 and *At*NDT2 have overlapping functions in NAD^+^ transport in different tissues and conditions. It will also be interesting to determine whether specific stress situations characterized by differential expression patterns of *AtNDT1* and *AtNDT2* correlated with the establishment or alteration of the mitochondrial pyridine nucleotide pool. It seems reasonable to assume that the generation of multiple mutants for NAD^+^ transport proteins will be needed to gain precise mechanistic insight into these phenotypes. Moreover, the determination of NAD^+^ and NADP^+^ levels at tissue‐specific and subcellular levels in these mutants is likely to enhance our knowledge of the specificities and redundancies of these different albeit partially complementary transport proteins allowing us to truly understand the physiological hierarchy under which they operate.

## Experimental procedures

### Plant material

The *ndt1* ^*−*^ *:ndt1* ^*−*^ line, previously named GK‐241G12, was obtained from the GABI‐KAT collection. This mutant line harbours a T‐DNA insertion in the ninth exon of *AtNDT1* (*At*2g47490) (Figure 1a). The mutant line was selected on medium containing sulfadiazine (https://www.sigmaaldrich.com) and the insertion of T‐DNA and homozygous plants were confirmed by PCR using specific primers for the gene that confers resistance to sulfadiazine (forward 5′‐GCACGAGGTACAAACCTCTACTCT‐3′, reverse 5′‐GTCTCTCAAGTTTCAACCCATTCT‐3′ and T‐DNA 5′‐ATATTGACCATCATACTCATTGC‐3′).

In addition, transgenic plants were generated by insertion of an antisense construct, under the control of the *35S* promoter, produced using the Gateway system (Landy, 1989). For this purpose, primers were designed (forward 5′‐CCACCATGTCCGCTAATTCTCATCCTC‐3′ and reverse 5′‐CTTAAAGTATAGAGCTTTGCTCAGAAGG‐3′) from a cDNA library, for amplification of the 939‐bp *AtNDT1* cDNA fragment. The PCR product was recombined with the vector donor pDONR207 generating an entry clone in *Escherichia coli* (DH5α strain). A second recombination was performed in pK2WG7 and transformed into *E. coli*. Subsequently, the selected colonies were used to transform *A. tumefaciens* (strain GV3101), which was then used for insertion in Arabidopsis plants, ecotype Columbia‐0, in which the transgene expression was driven by the constitutive 35S promoter (Bechtold *et al*., 1993). The cassette contained a marker gene, conferring resistance to hygromycin (https://www.thermofisher.com) driven by the *nos* promoter and *nos* terminator.

### Growth conditions

Seeds were surface sterilized and germinated on half‐strength Murashige and Skoog (½MS) medium (Murashige and Skoog, 1962), supplemented with 1% sucrose (w/v) and the selective agent corresponding to the genotype used. Seeds were stratified for 4 days at 4°C in the darkness and then kept in a growth chamber at 22 ± 2°C, 60% relative humidity, irradiance of 150 μmol photons m^−2^ sec^−1^ and a photoperiod of 8 h light and 16 h dark for 10 days. After, seedlings were transferred to pots containing 0.08 dm^3^ of commercial substrate, Tropstrato HT^®^, and maintained under the same conditions. During the 4^th^ week after transplanting, physiological assessments and harvesting of samples in liquid nitrogen for biochemical analyses were performed.

For the analyses involving heterotrophic tissues, plates were kept in an air‐conditioned growth room at 22 ± 2°C, relative humidity 60%, and an irradiance of 150 μmol photons m^−2^ sec^−1^, with a photoperiod of 8 h light and 16 h dark for 10 days. Then, the seedlings were also transferred to commercial substrate and evaluated for seed production.

### Subcellular localization of NDT1 and NDT2 by confocal laser scanning microscopy (CLSM)

Arabidopsis plants were stable transformed by *Agrobacterium‐*mediated transformation with NDT1 or NDT2 fused to a C‐terminal GFP tag under the control of the ubiquitin 10 promotor (Grefen *et al*., 2010). Five‐day‐old transformed seedlings were incubated in 200 nm Mito tracker Red CMX ROs in ½MS medium for 1 h in the dark. Afterwards, seedlings were washed with ½MS medium to remove the Mito tracker solution and analyzed by a CLSM (Zeiss LSM 780) and Zeiss Zen software. Excitation and emission ranges of GFP (488 nm/490–550 nm) and Mito tracker Red CMX ROs (561 nm/580–625 nm) were measured in two tracks. Image processing was performed using Fiji as previously described (Schindelin *et al*., 2012).

### Gene expression analysis

Gene expression analysis was performed in leaves, flowers, pollen, and imbibed seeds using quantitative real‐time PCR (qRT‐PCR). Total RNA was extracted from leaves and flowers using TRIzol^®^ reagent (https://www.thermofisher.com) according to the manufacturer's instructions. Then total RNA was treated with DNase/RNase‐free (https://www.thermofisher.com) and quantified spectrophotometrically at 260 nm. Approximately 2 μg of isolated RNA were used to synthesize the complementary strand of DNA (cDNA) using an Improm‐II^™^ Reverse Transcription System (Promega, Madison, WI, USA, https://www.promega.com) and oligo(dT)_15_, following the manufacturer's recommendations.

The pollen isolation was made using a liquid pollen collection method as described previously (Hicks *et al*., 2004), with some modifications. Briefly, 50 inflorescences were collected into cold pollen growth medium (PGM) 17% sucrose, 2 mm CaCl_2_, 1.625 mm boric acid pH adjusted to 7.5 with KOH. The pollen was released by agitating with gloved hands. After removing all green plant material the PGM was filtered in a nylon membrane (80 μm mesh size). Finally, after centrifugation at 5000 ***g*** for 10 min the pellet containing the pollen was immediately frozen until RNA extraction.

Total RNA from pollen and 48 h imbided seeds were both isolated using the SV Total RNA Isolation System (Promega, https://www.promega.com) following the manufacturer's manual. The integrity of the RNA was checked on 1% (w/v) agarose gels, and the concentration was measured using the QIAxpert system (QIAGEN, https://www.qiagen.com). Subsequently, total RNA was reverse transcribed into cDNA using the Universal RiboClone^®^ cDNA Synthesis System (Promega) according the manufacturer's protocol.

Quantitative RT‐PCRs were performed using a 7300 Real‐Time System (Applied Biosystems, Foster City, CA, USA, https://www.thermofisher.com) and Power SYBR^®^ Green PCR Master Mix (https://www.thermofisher.com), following the manufacturer's recommendations. The relative expression levels were normalized using housekeeping genes (Table S3) and calculated using the ΔΔCt method. All primers used for qRT‐PCR were designed using the QuantPrime software (Messinger *et al*., 2006) and are listed in Table S3. qRT‐PCR cycles were set up as follows: 94°C for 10 min, 40 cycles of 94°C for 15 sec, 58°C for 15 sec and 72°C for 15 sec.

### Gas‐exchange and chlorophyll *a* fluorescence parameters

Gas‐exchange and chlorophyll *a* fluorescence parameters were evaluated 1 h after the onset of the light period by an infrared gas analyzer (IRGA) LI 6400XT (LI‐COR, Lincoln, NE, USA, https://www.licor.com/) with coupled fluorometer (6400‐40 LI‐LI‐COR Inc.). Light response curves of net CO_2_ assimilation were obtained using a 2 cm^2^ foliar chamber, 25°C temperature, CO_2_ concentration (*C*_a_) of 400 μmol CO_2_ mol^−1^ and irradiances (PPFD) of 0, 10, 25, 50, 100, 200, 400, 800, 1000 and 1200 μmol photons m^−2^ sec^−1^. The variables derived from the curves *A*/PPFD, such as compensation irradiance (*I*_c_), saturation irradiance (*I*_s_), light use efficiency (1/ɸ), and CO_2_ assimilation rate saturated by light (*A*_RFA_) were estimated from response curve settings to light by the non‐rectangular hyperbolic model (von Caemmerer, 2000).

Gas‐exchange parameters were evaluated at saturation, i.e. 400 μmol photons m^−2^ sec^−1^ (Figure S10). Photosynthesis per mass unit was estimated based on SLA and LN.

The photorespiration rate (*R*_i_) was estimated as: *R*_i_ = ((1/12) × (*J*_flu_ − (4 × (*A* + *R*_d_)))) where *J*_flu_ equivalent to ETR estimated by fluorescence parameters (Valentini *et al*., 1995). The instantaneous water use efficiency (*A*/*E*) and intrinsic water use efficiency (*A*/*g*_s_), where *E* stands for transpiration and *g*_s_ for stomatal conductance, were also calculated.

Dark respiration (*R*_d_) was determined after 1 h dark acclimation using the same IRGA system described above. The maximum quantum efficiency of photosystem II (*F*_v_/*F*_m_) was evaluated. After 1 h of dark acclimation, an irradiance of 0.03 μmol photons m^−2^ sec^−1^ was applied to determine the initial fluorescence (*F*_0_). To obtain maximal fluorescence (*F*_m_), a saturating pulse of 6000 μmol photons m^−2^ sec^−1^ was applied for 0.8 sec. The *F*_v_/*F*_m_ was then calculated as (*F*_m_ − *F*_0_)/*F*_m_. Furthermore, NPQ and ETR were estimated as described by DaMatta *et al*. (2002) and Lima *et al*. (2002).

### Biometric analysis

Following measurement of gas exchange and fluorescence, the whole plant was harvested and the following growth parameters were evaluated: rosette dry weight (RDW), RSDW, root/shoot ratio (RRS), RLA, SRA, LN, TLA, and SLA. RLA and TLA were determined using a digital image, in which the leaves were scanned (Hewlett Packard Scanjet G2410, Palo Alto, CA, USA) and the obtained images were processed with the aid of Rosette Tracker software (De Vylder *et al*., 2012). SRA and SLA were estimated using the formula: SRA (or SLA) = RLA (or TLA)/RDW.

### Stomatal density

The stomatal density was determined using epidermal prints, from the abaxial surface of fully expanded leaves according to von Groll *et al*. (2002). Six plants per genotype were printed and, for each epidermal print, 10 different regions were evaluated.

### Biochemical analyses

Six whole rosettes of each line were collected from 5‐week‐old plants and snap frozen in liquid nitrogen at 8, 12 or 16 h corresponding to the beginning, middle and end of the light period and 24 h and 8 h representing the middle and end of the dark period, respectively. Subsequently, samples were homogenized, and subjected to ethanol extraction as described by Gibon *et al*. (2004). Chlorophyll, nitrate, glucose, fructose and sucrose contents were quantified according to Sulpice *et al*. (2009) and Fernie *et al*. (2001), soluble amino acids as described by Gibon *et al*. (2004) and malate and fumarate as detailed by Nunes‐Nesi *et al*. (2007). In the insoluble fraction, the levels of starch and protein were determined according to Cross *et al*. (2006). The rates of starch biosynthesis ((starch concentration at the end of the light period – starch concentration at the beginning of the light period)/the number of hours of light) and starch degradation ((starch concentration at the end of the light period – concentration starch at the end of the dark period)/number of dark hours) were calculated. In addition, aliquots of approximately 25 mg of leaf samples were collected in the middle of the light period for the quantification of nucleotide NAD^+^, NADH, NADP^+^ and NADPH according to the protocol described by Schippers *et al*. (2008).

The metabolic profile was determined by gas chromatography time‐of‐flight mass spectrometry (GC‐TOF MS) according to the protocol described by Lisec *et al*. (2006). Metabolites were manually identified using the reference library mass spectra and retention indices from the Golm Metabolome Database (http://gmd.mpimp-golm.mpg.de; Kopka *et al*., 2005). Metabolite profiling data are reported following recommendations (Fernie *et al*., 2011).

Fatty acids of mature dried seeds and seedlings at 2, 4 or 6 days old were extracted and derivatized as described by Browse *et al*. (1986), and analyzed as methyl esters (FAMEs). The fatty acid profile was analyzed by GC‐TOF MS. Before derivatisation, fatty acid 17:0 was added as an internal standard to enable quantification. The identification of compounds was based on retention time and comparison of the mass spectra with reference spectra available on the NIST 08 and NIST 08s library database (National Institute of Standards and Technology, Babushok *et al*., 2007). The quantification was performed automatically by integrating the chromatographic peaks obtained (Bernhardt *et al*., 2012). The amounts of the 17:0 standard was used for the correction of inter‐sample variation and absolute quantification of fatty acids according to Hielscher *et al*. (2017). The percentage of each fatty acid was calculated relative to the total of all fatty acids for each line per time point.

### Morphological analysis

The viability of pollen grain was evaluated as described by Lorenzon and Almeida (1996). For this, dehisced anthers of different genotypes were gently dipped onto the surface of the microscope slide and the pollen grains released were transferred to the dye and evaluated under a light microscope. Six flowers from six plants from each line were analyzed, with unstained or deformed pollen grains being considered non‐viable. We additionally determined the length and diameter of siliques using a stereomicroscope. Six siliques from six plants of each line were photographed and measured and the length, diameter and number of seeds in each silique were determined. In addition, the 1000‐seed weight was also determined.

### Seed germination and seedling development

Seeds of *ndt1* ^*−*^ *:ndt1* ^*−*^ and WT plants were surface sterilized and germinated as described above. After 48 h in the light, the percentage of germination, GSI, percentage of normal and abnormal seedlings (including albino seedlings) and the ESI was determined. GSI and ESI were calculated by the sum of the number of germinated seeds (or normal seedlings) each day, divided by the number of days between sowing and germination, according to Maguire (1962). Six replicates of 50 seeds each were used for this evaluation.

### Statistical analysis

All the data are expressed as the mean ± standard error. Data were tested for significant differences (*P *≤* *0.05) using Student′s *t* test. All the statistical analyses were performed using the algorithm embedded into Microsoft Excel^®^ (Microsoft, Seattle).

## Accession numbers

*AtNDT1* (*At*2g47490); *AtNDT2* (*At*1g25380); *AtPXN* (*At*2g39970); *COBL11* (*At*4g27110); F‐box family protein (*At*5g15710); *AtNADK1* (*At*3g21070); *AtNADK2* (*At*1G21640); *AtNADK3* (*At*1G78590); *AtACTIN* (*At*2g37620); *AtPARP1* (*At*2g31320); *AtPARP2* (*At*4g02390); *AtNUDIX7* (*At*4g12720); *AtNIC1* (*At*2G22570); *AtNIC4* (*At*3g16190); *AtNAPRT1* (*At*4g36940); *AtNAPRT2* (*At*2g23420); *AtNADS* (*At*1g55090); *AtNMNAT* (*At*5g55810); *AtQPT* (*At*2g01350); *AtQS* (*At*5g50210); *AtAO* (*At*5g14760).

## Data statement

All data used for the analyses are available upon request or as Supporting Information and may be found in the online version of this article.

## Conflict of interest

The authors declare no conflict of interest.

## Author contributions

AF and ANN screened and genotyped the mutant line. MVP performed the cloning and plant transformation under supervision of TO; ISC screened the transgenic lines and performed most of the experiments under supervision of ANN; DBM, PFP, LC, EH and JACA performed complementary experiments and analyses; NL and TM‐A supervised ISC in the lipid analysis and LC in protein localization analysis; ANN and ISC designed the experiments and analyzed the data; ARF and ANN conceived the project and wrote the article with contributions of all the authors; PFP, HEN, FP, WLA, NL and APMW complemented the writing.

## Supporting information


**Figure S1.** Gene expression analysis of the NDT1 gene in different organs of *Arabidopsis thaliana* wild type plants.

**Figure S2.** Gene expression analysis of genes encoding NAD^+^ carriers (NDT1, NDT2 and PXN) in different organs of *Arabidopsis thaliana* wild type and *ndt1^−^:ndt1^−^* plants.

**Figure S3.** Gene expression analysis of genes encoding enzymes related to NAD^+^ metabolism in imbibed seeds and leaves of *Arabidopsis thaliana* mutants deficient in the expression of the mitochondrial NAD^+^ transporter (NDT1) and wild type (WT) plants.

**Figure S4.** Phenotypic analysis of *Arabidopsis thaliana* lines deficient in the expression of the mitochondrial NAD^+^ transporter (NDT1) and wild type (WT) plants.

**Figure S5.** Seed, seedling, germination and seedling establishment characterization of *Arabidopsis thaliana* mutant line deficient in the expression of the mitochondrial NAD^+^ transporter (NDT1) and wild type (WT) plants.

**Figure S6.** Fatty acid composition in seeds and seedling of *Arabidopsis thaliana* mutants deficient in the expression of the mitochondrial NAD^+^ transporter (NDT1) and wild type (WT) plants.

**Figure S7.** Phenotypic analysis of pollen grains stained with acetic carmine from *Arabidopsis thaliana* genotypes deficient in the expression of the mitochondrial NAD^+^ transporter (NDT1) and wild type plants.

**Figure S8.** Germination rate and tube growth of pollen grains from *Arabidopsis thaliana* genotype deficient in the expression of the mitochondrial NAD^+^ transporter (NDT1) and wild type (WT) plants.

**Figure S9.** eFP display of transcript accumulation patterns across a variety of Arabidopsis organs and treatments.

**Figure S10.** Gas‐exchange and chlorophyll *a* fluorescence parameters in leaves of 4‐week‐old *Arabidopsis thaliana* genotypes deficient in the expression of the mitochondrial NAD^+^ transporter (NDT1) and wild type (WT) plants.

**Figure S11.** Non‐photochemical quenching (NPQ) of 4‐week‐old *Arabidopsis thaliana* genotypes deficient in the expression of the mitochondrial NAD^+^ transporter (NDT1) and wild type (WT) plants under various light intensities.

**Figure S12.** Changes in the main nitrogen metabolites in leaves of 4‐week‐old *Arabidopsis thaliana* genotypes deficient in the expression of the mitochondrial NAD^+^ transporter (NDT1) and wild type (WT) plants.

**Figure S13.** Changes in chlorophyll content in leaves of 4‐week‐old *Arabidopsis thaliana* genotypes deficient in the expression of the mitochondrial NAD^+^ transporter (NDT1) and wild type (WT) plants.

**Table S1.** Parameters derived from photosynthetic light curve response (Figure S4a) of 4‐week‐old, short‐day grown *Arabidopsis thaliana* genotypes deficient in the expression of the mitochondrial NAD^+^ transporter (NDT1).

**Table S2.** Relative metabolite levels in leaves of 4‐week‐old, short‐day grown, *Arabidopsis thaliana* genotypes deficient in the expression of the mitochondrial NAD^+^ transporter (NDT1) and wild type (WT) plants.

**Table S3.** List of primers used in this work to perform qPCR analysis.

## References

[tpj14452-bib-0001] Agledal, L. , Niere, M. and Ziegler, M. (2010) The phosphate makes a difference: cellular functions of NADP. Redox Rep. 15, 2–10. 10.1179/174329210x12650506623122 20196923 PMC7067316

[tpj14452-bib-0002] Agrimi, G. , Russo, A. , Pierri, C.L. and Palmieri, F. (2012) The peroxisomal NAD^+^ carrier of *Arabidopsis thaliana* transports coenzyme A and its derivatives. J. Bioenerg. Biomembr. 44, 333–340. 10.1007/s10863-012-9445-0 22555559

[tpj14452-bib-0003] Babushok, V.I. , Linstrom, P.J. , Reed, J.J. , Zenkevich, I.G. , Brown, R.L. , Mallard, W.G. and Stein, S.E. (2007) Development of a database of gas chromatographic retention properties of organic compounds. J. Chromatogr. A, 1157, 414–421. 10.1016/j.chroma.2007.05.044 17543315

[tpj14452-bib-0004] Baud, S. , Boutin, J.P. , Miquel, M. , Lepiniec, L. and Rochat, C. (2002) An integrated overview of seed development in *Arabidopsis thaliana* ecotype WS. Plant Physiol. Biochem. 40, 151–160. 10.1016/s0981-9428(01)01350-x

[tpj14452-bib-0005] Bechtold, N. , Ellis, J. and Pelletier, G. (1993) In planta *Agrobacterium* mediated gene transfer by infiltration of adult *Arabidopsis thaliana* plants. C. R. Acad. III, 316, 1194–1199. 10.1007/978-3-642-79247-2_3 9664431

[tpj14452-bib-0006] Bedhomme, M. , Hoffmann, M. , McCarthy, E.A. , Gambonnet, B. , Moran, R.G. , Rébeillé, F. and Ravanel, S. (2005) Folate metabolism in plants: an Arabidopsis homolog of the mammalian mitochondrial folate transporter mediates folate import into chloroplasts. J. Biol. Chem. 280, 34823–34831. 16055441 10.1074/jbc.M506045200

[tpj14452-bib-0007] Bernhardt, K. , Wilkinson, S. , Weber, A.P.M. and Linka, N. (2012) A peroxisomal carrier delivers NAD^+^ and contributes to optimal fatty acid degradation during storage oil mobilization. Plant J. 69, 1–13. 10.1111/j.1365-313x.2011.04775.x 21895810

[tpj14452-bib-0008] Berrin, J.‐G. , Pierrugues, O. , Brutesco, C. , Alonso, B. , Montillet, J.‐L. , Roby, D. and Kazmaier, M. (2005) Stress induces the expression of *AtNADK‐1*, a gene encoding a NAD(H) kinase in *Arabidopsis thaliana* . Mol. Genet. Genomics, 273, 10–19. 10.1007/s00438-005-1113-1 15711971

[tpj14452-bib-0009] Bowsher, C.G. , Lacey, A.E. , Hanke, G.T. , Clarkson, D.T. , Saker, L.R. , Stulen, I. and Emes, M.J. (2007) The effect of Glc6P uptake and its subsequent oxidation within pea root plastids on nitrite reduction and glutamate synthesis. J. Exp. Bot. 58, 1109–1118. 10.1093/jxb/erl269 17220512

[tpj14452-bib-0010] Browse, J. , McCourt, P.J. and Somerville, C.R. (1986) Fatty acid composition of leaf lipids determined after combined digestion and fatty acid methyl ester formation from fresh tissue. Anal. Biochem. 152, 141–145. 10.1016/0003-2697(86)90132-6 3954036

[tpj14452-bib-0011] Cárdenas, L. , McKenna, S.T. , Kunkel, J.G. and Hepler, P.K. (2006) NAD(P)H oscillates in pollen tubes and is correlated with tip growth. Plant Physiol. 142, 1460–1468. 10.1104/pp.109.150458 17041030 PMC1676060

[tpj14452-bib-0012] Chai, M.F. , Chen, Q.J. , An, R. , Chen, Y.M. , Chen, J. and Wang, X.C. (2005) NADK2, an Arabidopsis chloroplastic NAD kinase, plays a vital role in both chlorophyll synthesis and chloroplast protection. Plant Mol. Biol. 59, 553–564. 10.1007/s11103-005-6802-y 16244906

[tpj14452-bib-0013] Chai, M.F. , Wei, P.C. , Chen, Q.J. , An, R. , Chen, J. , Yang, S. and Wang, X.C. (2006) NADK3, a novel cytoplasmic source of NADPH, is required under conditions of oxidative stress and modulates abscisic acid responses in Arabidopsis. Plant J. 47, 665–674. 10.1111/j.1365-313x.2006.02816.x 16856986

[tpj14452-bib-0014] Cross, J.M. , von Korff, M. , Altmann, T. , Bartzetko, L. , Sulpice, R. , Gibon, Y. , Palacios, N. and Stitt, M. (2006) Variation of enzyme activities and metabolite levels in 24 Arabidopsis accessions growing in carbon‐limited conditions. Plant Physiol. 142, 1574–1588. 10.1104/pp.106.086629 17085515 PMC1676042

[tpj14452-bib-0015] DaMatta, F.M. , Loos, R.A. , Silva, E.A. and Loureiro, M.E. (2002) Limitations to photosynthesis in *Coffea canephora* as a result of nitrogen and water availability. J. Plant Physiol. 159, 975–981. 10.1078/0176-1617-00807

[tpj14452-bib-0016] De Block, M. , Verduyn, C. , De Brouwer, D. and Cornelissen, M. (2005) Poly(ADP‐ribose) polymerase in plants affects energy homeostasis, cell death and stress tolerance. Plant J. 41, 95–106. 10.1111/j.1365-313x.2004.02277.x 15610352

[tpj14452-bib-0017] De Vylder, J. , Vandenbussche, F. , Hu, Y. , Philips, W. and Van Der Straeten, D. (2012) Rosette Tracker: an open source image analysis tool for automatic quantification of genotype effects. Plant Physiol. 160, 1149–1159. 10.1104/pp.112.202762 22942389 PMC3490612

[tpj14452-bib-0018] Fernie, A.R. , Roscher, A. , Ratcliffe, R.G. and Kruger, N.J. (2001) Fructose 2,6‐bisphosphate activates pyrophosphate: fructose 6 phosphate 1‐phosphotransferase and increases triose phosphate to hexose phosphate cycling in heterotrophic cells. Planta, 212, 250–263. 10.1007/s004250000386 11216846

[tpj14452-bib-0019] Fernie, A.R. , Aharoni, A. , Willmitzer, L. , Stitt, M. , Tohge, T. , Kopka, J. , Carroll, A.J. , Saito, K. , Fraser, P.D. and de Luca, V. (2011) Recommendations for reporting metabolite data. Plant Cell, 23, 2477–2482. 10.1105/tpc.111.086272 21771932 PMC3226225

[tpj14452-bib-0020] Flexas, J. , Ribas‐Carbó, M. , Diaz‐Espejo, A. , Galmés, J. and Medrano, H. (2008) Mesophyll conductance to CO_2_: current knowledge and future prospects. Plant Cell Environ. 31, 602–621. 10.1111/j.1365-3040.2007.01757.x 17996013

[tpj14452-bib-0021] Gakière, B. , Fernie, A.R. and Pétriacq, P. (2018) More to NAD^+^ than meets the eye: A regulator of metabolic pools and gene expression in Arabidopsis. Free Radical Biology and Medicine, 122, 86–95. 10.1016/j.freeradbiomed.2018.01.003 29309893

[tpj14452-bib-0022] Geigenberger, P. and Fernie, A.R. (2014) Metabolic control of redox and redox control of metabolism in plants. Antioxid. Redox Signal. 21, 1389–1421. 10.1089/ars.2014.6018 24960279 PMC4158967

[tpj14452-bib-0023] Geigenberger, P. , Kolbe, A. and Tiessen, A. (2005) Redox regulation of carbon storage and partitioning in response to light and sugars. J. Exp. Bot. 56, 1469–1479. 10.1093/jxb/eri178 15863446

[tpj14452-bib-0024] Gibon, Y. , Blaesing, O.E. , Hannemann, J. , Carillo, P. , Hohne, M. , Hendriks, J.H.M. , Palacios, N. , Cross, J. , Selbig, J. and Stitt, M. (2004) A robot‐based platform to measure multiple enzyme activities in Arabidopsis using a set of cycling assays: comparison of changes of enzyme activities and transcript levels during diurnal cycles and prolonged darkness. Plant Cell, 16, 3304–3325. 10.1105/tpc.104.025973 15548738 PMC535875

[tpj14452-bib-0025] Grefen, C. , Donald, N. , Hashimoto, K. , Kudla, J. , Schumacher, K. and Blatt, M.R. (2010) A ubiquitin-10 promoter-based vector set for fluorescent protein tagging facilitates temporal stability and native protein distribution in transient and stable expression studies. The Plant Journal, 64(2), 355–365. 10.1111/j.1365-313X.2010.04322.x 20735773

[tpj14452-bib-0026] Hashida, S.N. , Takahashi, H. , Kawai‐Yamada, M. and Uchimiya, H. (2007) *Arabidopsis thaliana* nicotinate/nicotinamide mononucleotide adenyltransferase (*At*NMNAT) is required for pollen tube growth. Plant J. 49, 694–703. 10.1111/j.1365-313x.2006.02989.x 17270012

[tpj14452-bib-0027] Hashida, S.N. , Takahashi, H. and Uchimiya, H. (2009) The role of NAD biosynthesis in plant development and stress responses. Ann. Bot. 103, 819–824. 10.1093/aob/mcp019 19201765 PMC2707885

[tpj14452-bib-0028] Hashida, S.N. , Itami, T. , Takahashi, H. , Takahara, K. , Nagano, M. , Kawai‐Yamada, M. , Shoji, K. , Goto, F. , Yoshihara, T. and Uchimiya, H. (2010) Nicotinate/nicotinamide mononucleotide adenyltransferase‐mediated regulation of NAD biosynthesis protects guard cells from reactive oxygen species in ABA‐mediated stomatal movement in Arabidopsis. J. Exp. Bot. 61, 3813–3825. 10.1093/jxb/erq190 20591898

[tpj14452-bib-0029] Hashida, S.N. , Takahashi, H. , Takahara, K. , Kawai‐Yamada, M. , Kitazaki, K. , Shoji, K. , Goto, F. , Yoshihara, T. and Uchimiya, H. (2013) NAD^+^ accumulation during pollen maturation in Arabidopsis regulating onset of germination. Mol. Plant, 6, 216–225. 10.1093/mp/sss071 22907882

[tpj14452-bib-0030] Hicks, G.R. , Rojo, E. , Hong, S. , Carter, D.G. and Raikhel, N.V. (2004) Geminating pollen has tubular vacuoles, displays highly dynamic vacuole biogenesis, and requires *VACUOLESS1* for proper function. Plant Physiol. 134, 1227–1239. 10.1104/pp.103.037382 14988481 PMC389947

[tpj14452-bib-0031] Hielscher, B. , Charton, L. , Mettler‐Altmann, T. and Linka, N. (2017) Analysis of Peroxisomal beta‐oxidation during storage oil mobilization in *Arabidopsis thaliana* seedlings. Methods Mol. Biol. 1595, 291–304. 10.1007/978-1-4939-6937-1_27 28409472

[tpj14452-bib-0032] Hölscher, C. , Lutterbey, M.C. , Lansing, H. , Meyer, T. , Fischer, K. and von Schaewen, A. (2016) Defects in peroxisomal 6‐phosphogluconate dehydrogenase isoform PGD2 prevent gametophytic interaction in *Arabidopsis thaliana* . Plant Physiol. 171, 192–205. 10.1104/pp.15.01301 26941195 PMC4854672

[tpj14452-bib-0033] Hunt, L. and Gray, J.E. (2009) The relationship between pyridine nucleotides and seed dormancy. New Phytol. 181, 62–70. 10.1111/j.1469-8137.2008.02641.x 18826484

[tpj14452-bib-0034] Hunt, L. , Holdsworth, M.J. and Gray, J.E. (2007) Nicotinamidase activity is important for germination. Plant J. 51, 341–351. 10.1111/j.1365-313x.2007.03151.x 17587307

[tpj14452-bib-0035] Kirchberger, S. , Tjaden, J. and Neuhaus, H.E. (2008) Characterization of the Arabidopsis Brittle1 transport protein and impact of reduced activity on plant metabolism. Plant J. 56, 51–63. 10.1111/j.1365-313x.2008.03583.x 18564385

[tpj14452-bib-0036] Kopka, J. , Schauer, N. , Krueger, S. ***et al.*** (2005) GMD@CSB.DB: the Golm Metabolome Database. Bioinformatics, 21, 1635–1638. 10.1093/bioinformatics/bti236 15613389

[tpj14452-bib-0037] Landy, A. (1989) Dynamic, structural, and regulatory aspects of Lambda site specific recombination. Ann. Rev. Biochem. 58, 913–949. 10.1146/annurev.bi.58.070189.004405 2528323

[tpj14452-bib-0038] Lima, A.L.S. , DaMatta, F.M. , Pinheiro, H.A. , Totola, M.R. and Loureiro, M.E. (2002) Photochemical responses and oxidative stress in two clones of *Coffea canephora* under water deficit conditions. Environ. Exp. Bot. 47, 239–247. 10.1016/s0098-8472(01)00130-7

[tpj14452-bib-0039] Lisec, J. , Schauer, N. , Kopka, J. , Willmitzer, L. and Fernie, A.R. (2006) Gas chromatography mass spectrometry‐based metabolite profiling in plants. Nat. Protoc. 1, 387–396. 10.1038/nprot.2006.59 17406261

[tpj14452-bib-0040] Liu, Y.‐J. , Nunes‐Nesi, A. , Wallström, S.V. , Lager, I. , Michalecka, A.M. , Norberg, F.E.B. , Widell, S. , Fredlund, K.M. , Fernie, A.R. and Rasmusson, A.G. (2009) A redox‐mediated modulation of stem bolting in transgenic nicotiana sylvestris differentially expressing the external mitochondrial NADPH dehydrogenase. Plant Physiol. 150, 1248–1259. 10.1104/pp.109.136242 19429607 PMC2705030

[tpj14452-bib-0041] Lorenzon, M.C.A. and Almeida, E.C. (1996) Viabilidade e germinação do pólen de linhagens parentais de cebola híbrida. Pesqui. Agropec. Bras. 32, 345–349

[tpj14452-bib-0042] Maguire, J.D. (1962) Speed of germination aid in selection and evaluation for seedling emergence and vigor. Crop Sci. 2, 176–177. 10.2135/cropsci1962.0011183x000200020033x

[tpj14452-bib-0043] Messinger, S.M. , Buckley, T.N. and Mott, K.A. (2006) Evidence for involvement of photosynthetic processes in the stomatal response to CO_2_ . Plant Physiol. 140, 771–778. 10.1104/pp.105.073676 16407445 PMC1361342

[tpj14452-bib-0044] Muñoz‐Bertomeu, J. , Cascales‐Minana, B. , Irles‐Segura, A. , Mateu, I. , Nunes‐Nesi, A. , Fernie, A.R. , Segura, J. and Ros, R. (2010) The plastidial glyceraldehyde‐3‐phosphate dehydrogenase is critical for viable pollen development in Arabidopsis. Plant Physiol. 152, 1830–1841 20107025 10.1104/pp.109.150458 PMC2850016

[tpj14452-bib-0045] Murashige, T. and Skoog, F. (1962) A revised medium for rapid growth and bioassay with tobacco tissue cultures. Physiol. Plant. 15, 473–497. 10.1111/j.1399-3054.1962.tb08052.x

[tpj14452-bib-0046] Neuhaus, H.E. and Emes, M.J. (2000) Nonphotosynthetic metabolism in plastids. Annu. Rev. Plant Physiol. Plant Mol. Biol. 51, 111–140. 10.1146/annurev.arplant.51.1.111 15012188

[tpj14452-bib-0047] Noctor, G. , Queval, G. and Gakière, B. (2006) NAD(P) synthesis and pyridine nucleotide cycling in plants and their potential importance in stress conditions. J. Exp. Bot. 57, 1603–1620. 10.1093/jxb/erj202 16714307

[tpj14452-bib-0048] Nunes‐Nesi, A. , Carrari, F. , Gibon, Y. , Sulpice, R. , Lytovchenko, A. , Fisahn, J. , Graham, J. , Ratcliffe, R.G. , Sweetlove, L.J. and Fernie, A.R. (2007) Deficiency of mitochondrial fumarase activity in tomato plants impairs photosynthesis via an effect on stomatal function. Plant J. 50, 1093–1106. 10.1111/j.1365-313x.2007.03115.x 17461782

[tpj14452-bib-0049] Palmieri, F. , Rieder, B. , Ventrella, A. ***et al.*** (2009) Molecular identification and functional characterization of *Arabidopsis thaliana* mitochondrial and chloroplastic NAD^+^ carrier proteins. J. Biol. Chem. 284, 31249–31259. 10.1074/jbc.m109.041830 19745225 PMC2781523

[tpj14452-bib-0050] Penfound, T. and Foster, J.W. (1999) NAD‐dependent DNA‐binding activity of the bifunctional NadR regulator of *Salmonella typhimurium* . J. Bacteriol. 181, 648–655. 9882682 10.1128/jb.181.2.648-655.1999 PMC93422

[tpj14452-bib-0051] Scheibe, R. (2004) Malate valves to balance cellular energy supply. Physiol. Plant. 120, 21–26. 10.1111/j.0031-9317.2004.0222.x 15032873

[tpj14452-bib-0052] Schippers, J.H.M. , Nunes‐Nesi, A. , Apetrei, R. , Hille, J. , Fernie, A.R. and Dijkwel, P.P. (2008) The *Arabidopsis* onset of leaf death5 mutation of quinolinate synthase nicotinamide adenine dinucleotide biosynthesis and causes early ageing. Plant Cell, 20, 2909–2925. 10.1105/tpc.107.056341 18978034 PMC2590718

[tpj14452-bib-0053] Selinski, J. and Scheibe, R. (2014) Pollen tube growth: where does the energy come from? Plant Signal. Behav. 9, e977200–e977209. 10.4161/15592324.2014.977200 25482752 PMC4622831

[tpj14452-bib-0054] Selinski, J. , König, N. , Wellmeyer, B. , Hanke, G.T. , Linke, V. , Neuhaus, H.E. and Scheibe, R. (2014) The plastid‐localized NAD‐dependent malate dehydrogenase is crucial for energy homeostasis in developing *Arabidopsis thaliana* seeds. Mol. Plant, 7, 170–186 24198233 10.1093/mp/sst151

[tpj14452-bib-0055] Senkler, J. , Senkler, M. , Eubel, H. , Hildebrandt, T. , Lengwenus, C. , Schertl, P. , Schwarzländer, M. , Wagner, S. , Wittig, I. and Braun, H.P. (2017) The mitochondrial complexome of *Arabidopsis thaliana* . Plant J. 89, 1079–1092. [Epub 2017 Feb 20]. 10.1111/tpj.13448 27943495

[tpj14452-bib-0056] Schindelin, J. , Arganda‐Carreras, I. , Frise, E. ***et al.*** (2012) Fiji: an open‐source platform for biological‐image analysis. Nat. Methods, 9, 676–682 22743772 10.1038/nmeth.2019 PMC3855844

[tpj14452-bib-0057] Sulpice, R. , Pyl, E.T. , Ishihara, H. ***et al.*** (2009) Starch as a major integrator in the regulation of plant growth. Proc. Natl Acad. Sci. USA, 106, 10348–10353. 10.1073/pnas.0903478106 19506259 PMC2693182

[tpj14452-bib-0058] Takahashi, H. , Takahara, K. , Hashida, S. , Hirabayashi, T. , Fujimori, T. , Kawai‐Yamada, M. , Yamaya, T. , Yanagisawa, S. and Uchimiya, H. (2009) Pleiotropic modulation of carbon and nitrogen metabolism in Arabidopsis plants overexpressing the *NAD kinase2* Gene. Plant Physiol. 151, 100–113. 10.1104/pp.109.140665 19587098 PMC2735975

[tpj14452-bib-0059] Takahashi, H. , Watanabe, A. , Tanaka, A. , Hashida, S. , Kawai‐yamada, M. , Sonoike, K. and Uchimiya, H. (2006) Chloroplast NAD Kinase is essential for energy transduction through the xanthophyll cycle in photosynthesis. Plant Cell Physiol. 47, 1678–1682 17082216 10.1093/pcp/pcl029

[tpj14452-bib-0060] Tanaka, Y. , Sugano, S.S. , Shimada, T. and Hara‐Nishimura, I. (2013) Enhancement of leaf photosynthetic capacity through increased stomatal density in Arabidopsis. New Phytol. 198, 757–764. 10.1111/nph.12186 23432385

[tpj14452-bib-0061] Todisco, S. , Agrimi, G. , Castegna, A. and Palmieri, F. (2006) Identification of the mitochondrial NAD1 transporter in *Saccharomyces cerevisiae* . J. Biol. Chem. 281, 1524–1531. 10.1074/jbc.m510425200 16291748

[tpj14452-bib-0062] Turner, W.L. , Waller, J.C. and Snedden, W.A. (2005) Identification, molecular cloning and functional characterization of a novel NADH kinase from *Arabidopsis thaliana* (thale cress). Biochem. J. 385, 217–223. 10.1042/bj20040292 15347288 PMC1134690

[tpj14452-bib-0063] Valentini, R. , Epron, D. , De Angelis, P. , Matteucci, G. and Dreyer, E. (1995) *In situ* estimation of net CO_2_ assimilation, photosynthetic electron flow and photorespiration in Turkey oak (*Q. cerris* L.) leaves: diurnal cycles under different levels of water supply. Plant Cell Environ. 18, 631–640. 10.1111/j.1365-3040.1995.tb00564.x

[tpj14452-bib-0064] VanLinden, M.R. , Dölle, C. , Pettersen, I.K. ***et al.*** (2015) Subcellular distribution of NAD^+^ between cytosol and mitochondria determines the metabolic profile of human cells. J. Biol. Chem. 290, 27644–27659. 10.1074/jbc.m115.654129 26432643 PMC4646015

[tpj14452-bib-0065] van Roermund, C.W. , Schroers, M.G. , Wiese, J. ***et al.*** (2016) The peroxisomal NAD carrier from Arabidopsis imports NAD in exchange with AMP. Plant Physiol. 171, 2127–2139 27208243 10.1104/pp.16.00540 PMC4936582

[tpj14452-bib-0066] , von Caemmerer, S. (2000). Biochemical Models of Leaf Photosynthesis. Victoria: CSIRO Publishing.

[tpj14452-bib-0067] von Groll, U. , Berger, D. and Altmann, T. (2002) The subtilisin‐like serine protease SDD1 mediates cell‐to‐cell signaling during Arabidopsis stomatal development. Plant Cell, 14, 1527–1539. 10.1105/tpc.001016 12119372 PMC150704

[tpj14452-bib-0068] Waller, J.C. , Dhanoa, P.K. , Schumann, U. , Mullen, R.T. and Snedden, W.A. (2010) Subcellular and tissue localization of NAD kinases from Arabidopsis: compartmentalization of de novo NADP biosynthesis. Planta, 231, 305–317. 10.1007/s00425-009-1047-7 19921251

[tpj14452-bib-0069] Winter, D. , Vinegar, B. , Nahal, H. , Ammar, R. , Wilson, G.V. and Provart, N.J. (2007) An “Electronic Fluorescent Pictograph” browser for exploring and analyzing large‐scale biological data sets. PLoS ONE, 2, e718 17684564 10.1371/journal.pone.0000718 PMC1934936

