# Supplementary material for: The mitochondrial NAD + transporter (NDT1) plays important roles in cellular NAD + homeostasis in Arabidopsis thaliana
Source: Plant J. 2019 Aug 9;100(3):487–504. doi: 10.1111/tpj.14452 (PMC6900047; doi:10.1111/tpj.14452)
Supplement: Supplementary file 1 — Figure S1. Gene expression analysis of the NDT1 gene in different organs of Arabidopsis thaliana wild type plants. [file TPJ-100-487-s001.pdf]

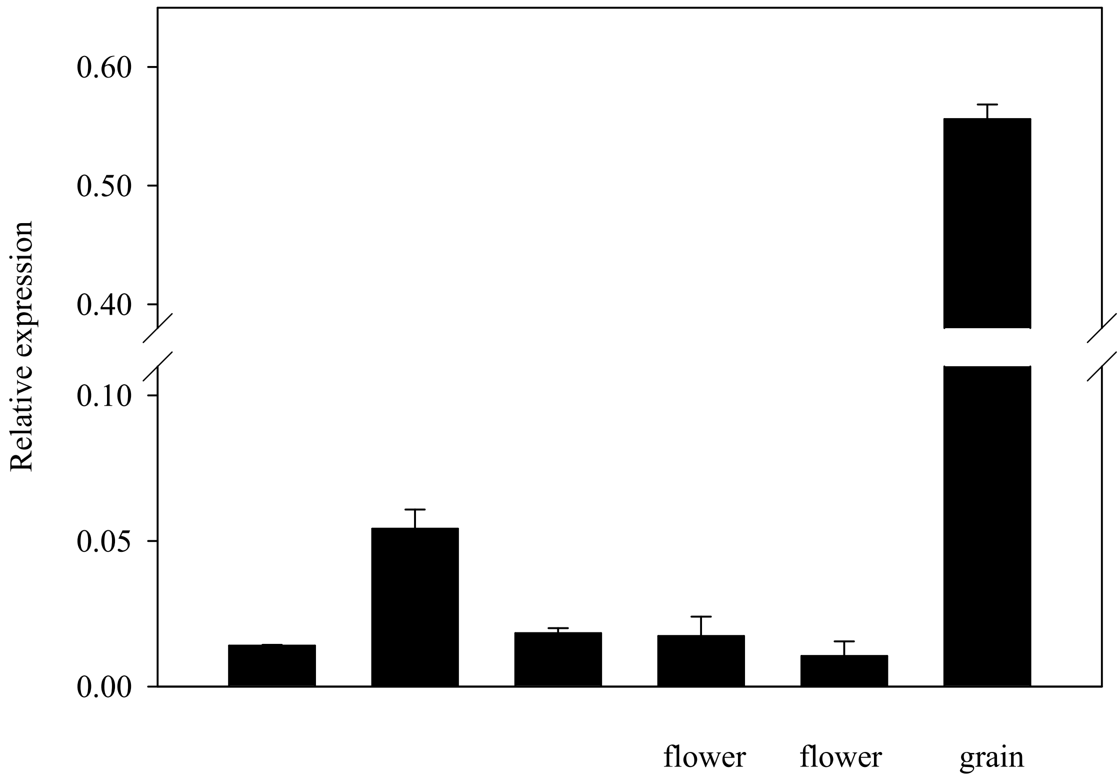

Figure S1. Gene expression analysis of the *NDT1* gene in different organs of *Arabidopsis thaliana* wild type plants.

Relative transcript abundance was determined by qRT-PCR from samples of wild type plants in different stages of development (48 h imbibed seeds, 8 days-old seedlings, mature leaves from 28 days-old plants, closed (Day 0) and open (Day 4) flowers, and pollen grains. Relative abundance of transcripts was calculated as $2^{-\Delta Ct}$ by subtracting the CT of the targets for the CT of the housekeeping gene ACT2. The values shown are means of three repeats (indicated by error bars).
