# Supplementary material for: The mitochondrial NAD + transporter (NDT1) plays important roles in cellular NAD + homeostasis in Arabidopsis thaliana
Source: Plant J. 2019 Aug 9;100(3):487–504. doi: 10.1111/tpj.14452 (PMC6900047; doi:10.1111/tpj.14452)
Supplement: Supplementary file 2 — Figure S2. Gene expression analysis of genes encoding NAD+ carriers (NDT1, NDT2 and PXN) in different organs of Arabidopsis thaliana wild type and ndt1−:ndt1− plants. [file TPJ-100-487-s002.pdf]

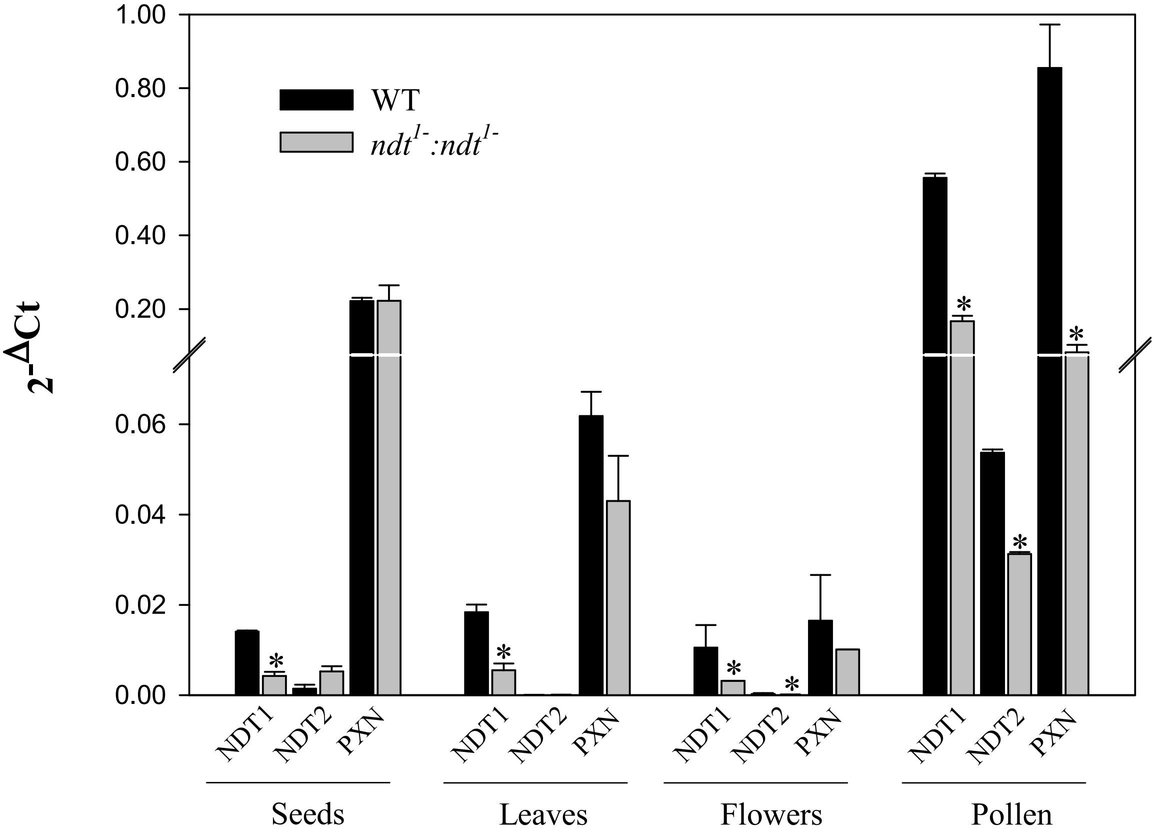

Figure S2. Gene expression analysis of genes encoding NAD⁺ carriers (NDT1, NDT2 and PXN) in different organs of *Arabidopsis thaliana* wild type and *ndt1⁻:ndt1⁻* plants.

The relative expression was determined by real-time quantitative PCR and calculated relative to the wild type (WT) in imbibed seeds, leaves from 28-days-old rosettes, open flowers (day 2) and pollen grains. Values are presented as mean ± SE of four individual plants per line; an asterisk indicates values that were determined by Student's *t* test to be significantly different ($P < 0.05$) from the WT.
