# Supplementary material for: The mitochondrial NAD + transporter (NDT1) plays important roles in cellular NAD + homeostasis in Arabidopsis thaliana
Source: Plant J. 2019 Aug 9;100(3):487–504. doi: 10.1111/tpj.14452 (PMC6900047; doi:10.1111/tpj.14452)
Supplement: Supplementary file 4 — Figure S4. Phenotypic analysis of Arabidopsis thaliana lines deficient in the expression of the mitochondrial NAD+ transporter (NDT1) and wild type (WT) plants. [file TPJ-100-487-s004.pdf]

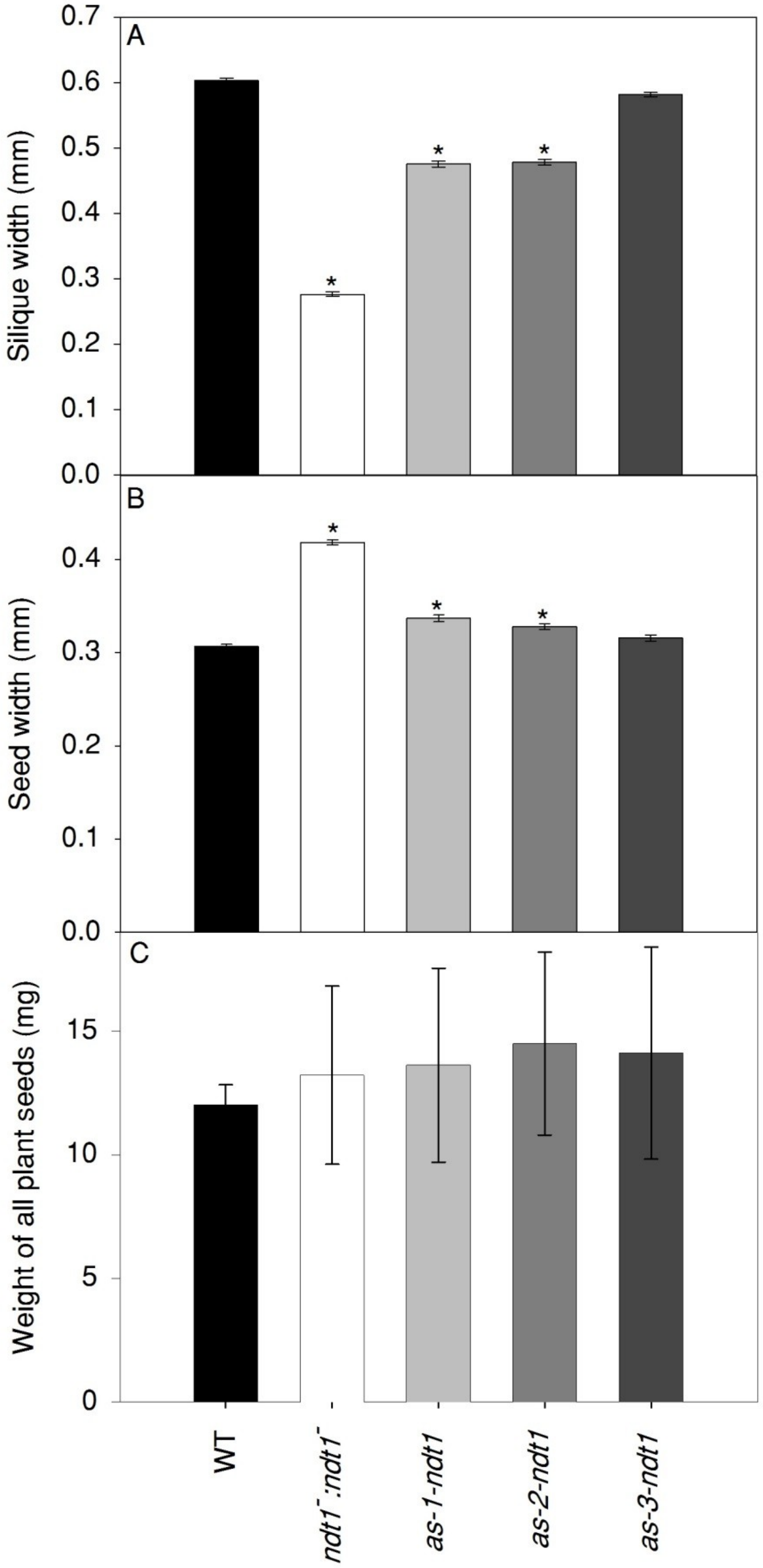

Figure S4. Phenotypic analysis of *Arabidopsis thaliana* lines deficient in the expression of the mitochondrial NAD$^+$ transporter (NDT1) and wild type (WT) plants. (A) Silique width. (B) Seed width. (C) Weight of all plant seeds. Values are presented as mean ± SE of six individual plants per line; an asterisk indicates values that were determined by Student's *t* test to be significantly different ($P < 0.05$) from the WT.
