# Supplementary material for: The mitochondrial NAD + transporter (NDT1) plays important roles in cellular NAD + homeostasis in Arabidopsis thaliana
Source: Plant J. 2019 Aug 9;100(3):487–504. doi: 10.1111/tpj.14452 (PMC6900047; doi:10.1111/tpj.14452)
Supplement: Supplementary file 5 — Figure S5. Seed, seedling, germination and seedling establishment characterization of Arabidopsis thaliana mutant line deficient in the expression of the mitochondrial NAD+ transporter (NDT1) and wild type (WT) plants. [file TPJ-100-487-s005.pdf]

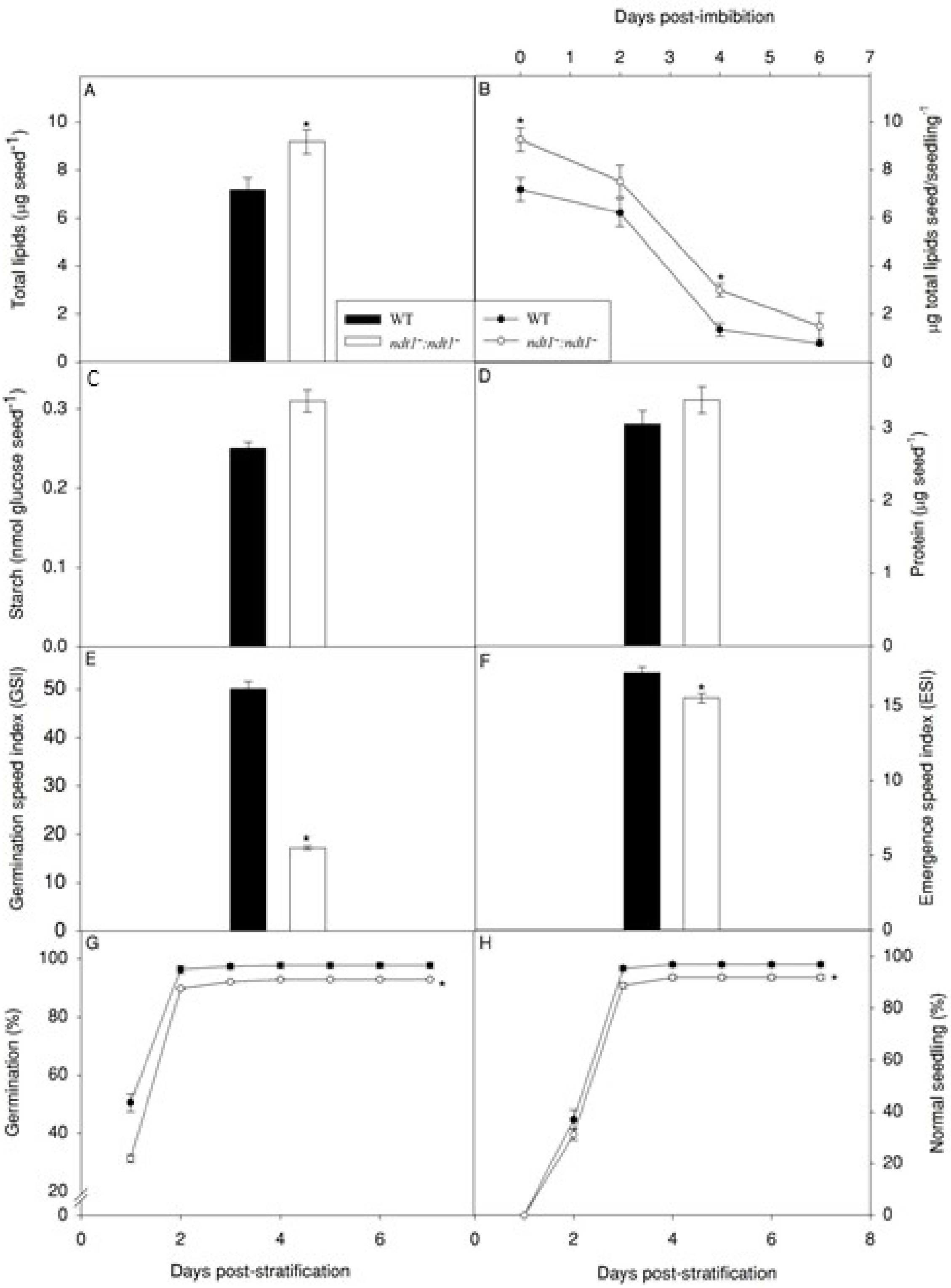

Figure S5. Seed, seedling, germination and seedling establishment characterization of *Arabidopsis thaliana* mutant line deficient in the expression of the mitochondrial NAD⁺ transporter (NDT1) and wild type (WT) plants. Values in figures A-D are presented as mean ± SE (n = 5) and in figures E-H are presented as mean ± SE of six individual plates with 50 seeds each per line; an asterisk indicates values that were determined by the Student's *t* test to be significantly different ($P < 0.05$) from the WT.
