# Supplementary material for: The mitochondrial NAD + transporter (NDT1) plays important roles in cellular NAD + homeostasis in Arabidopsis thaliana
Source: Plant J. 2019 Aug 9;100(3):487–504. doi: 10.1111/tpj.14452 (PMC6900047; doi:10.1111/tpj.14452)
Supplement: Supplementary file 6 — Figure S6. Fatty acid composition in seeds and seedling of Arabidopsis thaliana mutants deficient in the expression of the mitochondrial NAD+ transporter (NDT1) and wild type (WT) plants. [file TPJ-100-487-s006.pdf]

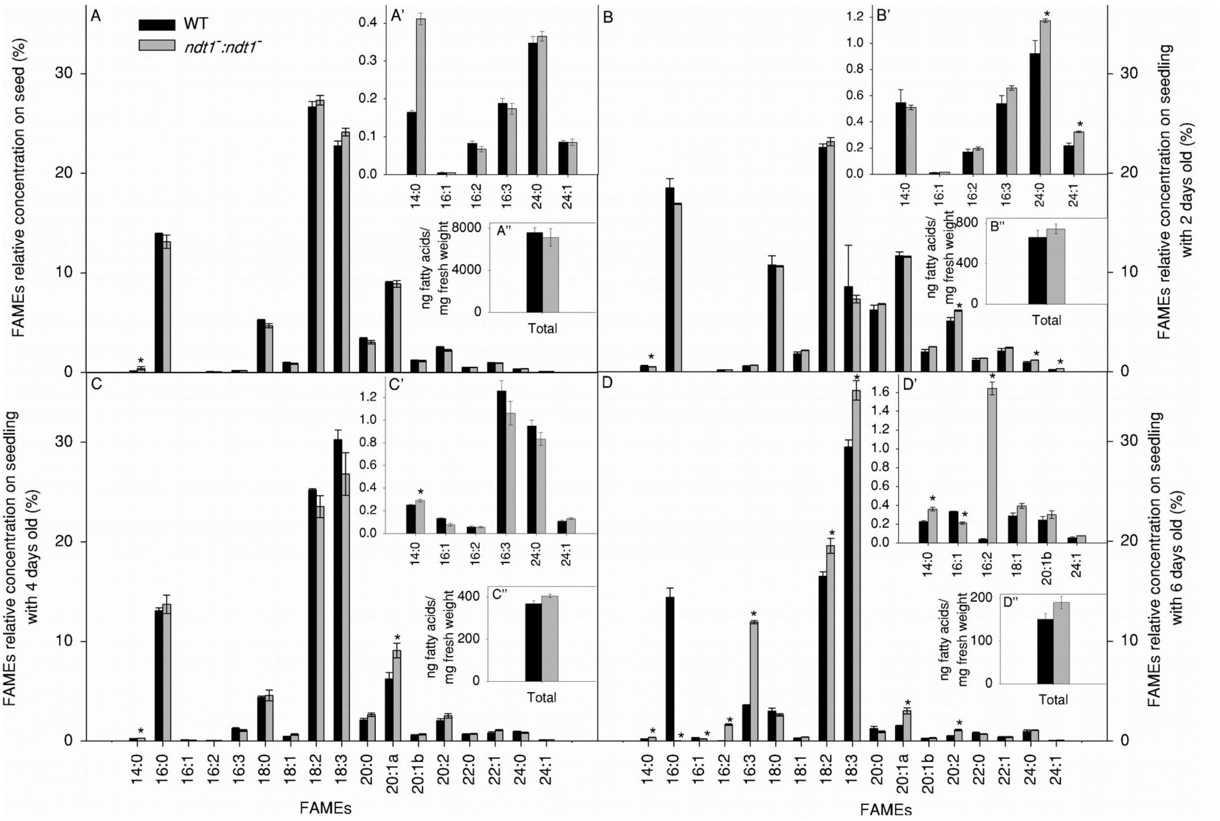

Figure S6. Fatty acid composition in seeds and seedling of *Arabidopsis thaliana* mutants deficient in the expression of the mitochondrial NAD$^+$ transporter (NDT1) and wild type (WT) plants. Fatty acid composition was analyzed by GC of fatty acid methyl esters (FAMEs). (A) Seeds; (B) 2-day-old seedlings; (C) 4-day-old seedlings; (D) 6-day-old seedlings. Detail presenting FAMEs for best visualization. Values are presented as mean ± SE of three individual samples per line; an asterisk indicates values that were determined by the Student's *t* test to be significantly different ($P < 0.05$) from the WT.
