# Supplementary material for: The mitochondrial NAD + transporter (NDT1) plays important roles in cellular NAD + homeostasis in Arabidopsis thaliana
Source: Plant J. 2019 Aug 9;100(3):487–504. doi: 10.1111/tpj.14452 (PMC6900047; doi:10.1111/tpj.14452)
Supplement: Supplementary file 7 — Figure S7. Phenotypic analysis of pollen grains stained with acetic carmine from Arabidopsis thaliana genotypes deficient in the expression of the mitochondrial NAD+ transporter (NDT1) and wild type plants. [file TPJ-100-487-s007.pdf]

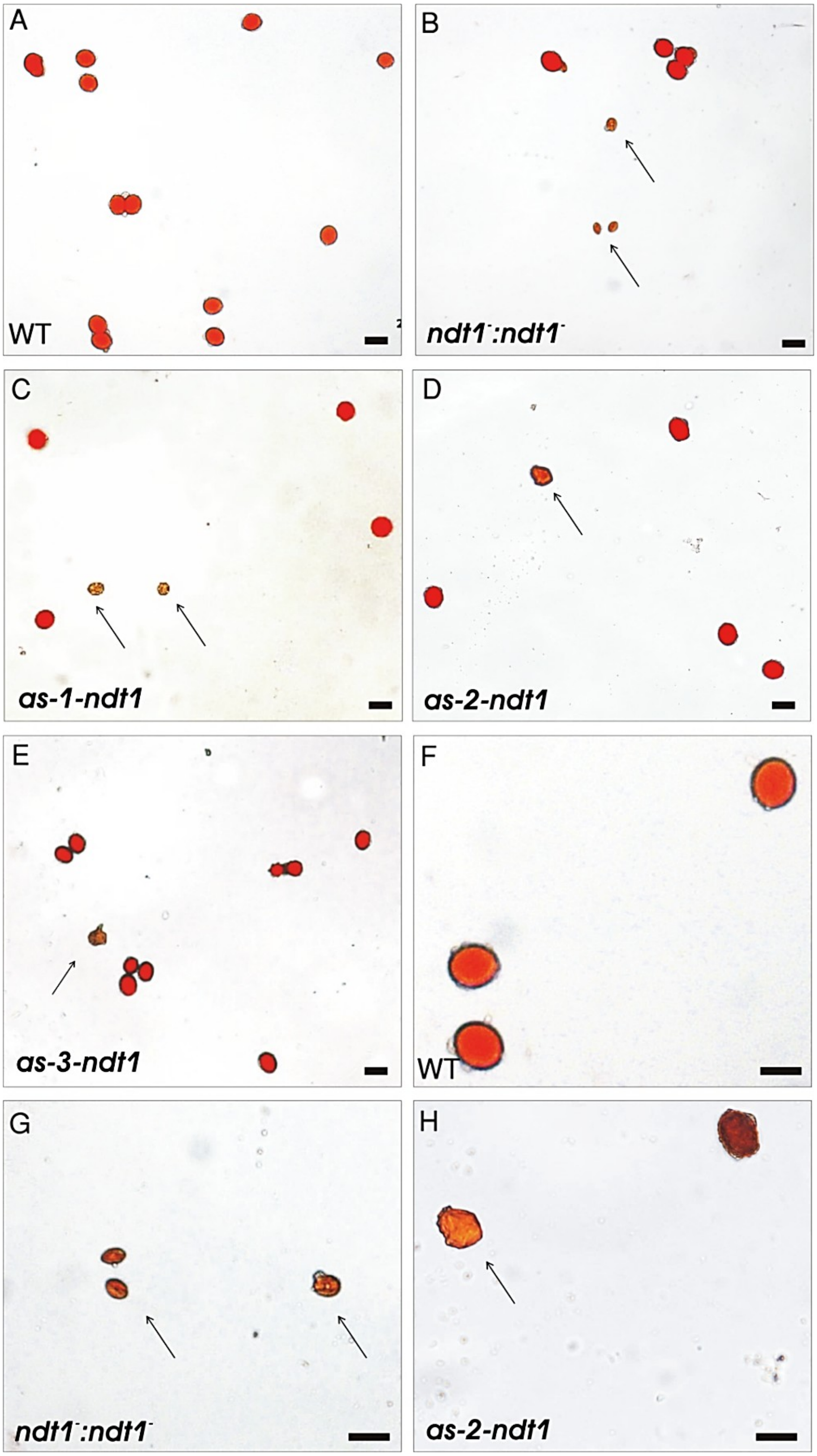

Figure S7. Phenotypic analysis of pollen grains stained with acetic carmine from *Arabidopsis thaliana* genotypes deficient in the expression of the mitochondrial NAD^+ transporter (NDT1) and wild type plants. Pollen grains stained with red were considered as viable and with yellow the non-viable ones (indicated with an arrow). Unstained or deformed pollen grains were also considered as non-viable. (A) Wild type (WT); (B) *ndt1⁻:ndt1⁻*; (C) *as-1-ndt1*; (D) *as-2-ndt1*; (E) *as-3-ndt1*; (F) detail of WT pollen grain; (G) detail *ndt1⁻:ndt1⁻* non-viable pollen grain (smaller than WT); (H) detail *as-2-ndt1* deformed pollen grain. Bars: 20 μm.
