# Supplementary material for: The mitochondrial NAD + transporter (NDT1) plays important roles in cellular NAD + homeostasis in Arabidopsis thaliana
Source: Plant J. 2019 Aug 9;100(3):487–504. doi: 10.1111/tpj.14452 (PMC6900047; doi:10.1111/tpj.14452)
Supplement: Supplementary file 8 — Figure S8. Germination rate and tube growth of pollen grains from Arabidopsis thaliana genotype deficient in the expression of the mitochondrial NAD+ transporter (NDT1) and wild type (WT) plants. [file TPJ-100-487-s008.pdf]

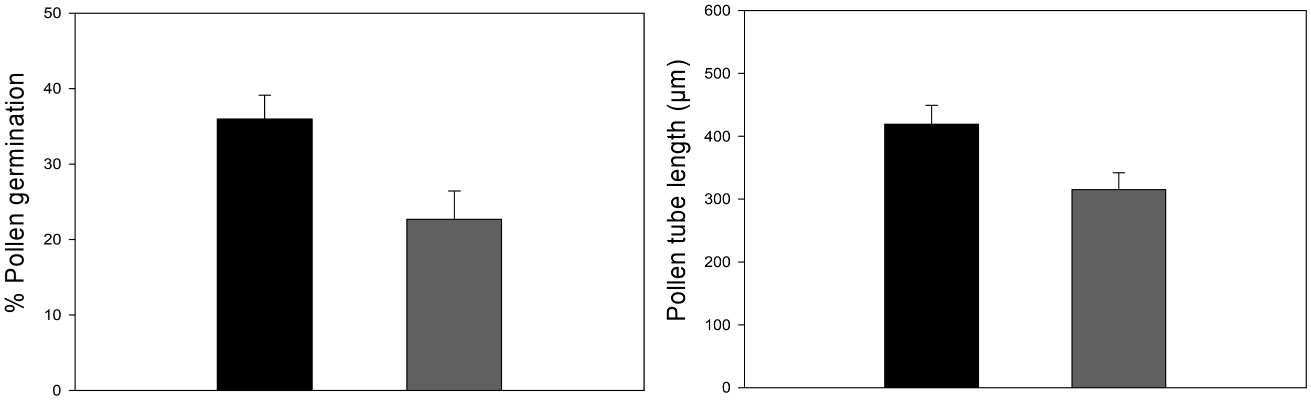

Figure S8. Germination rate and tube growth of pollen grains from *Arabidopsis thaliana* genotype deficient in the expression of the mitochondrial NAD$^+$ transporter (NDT1) and wild type (WT) plants. A) Percentage of pollen tube growth which represents germination/total number of grains analyzed. B) Pollen tube length. Pollen grains from 3 flowers per replication were placed immediately onto solidified pollen-germination medium and incubated at 22ºC for 8 hours. Bars represent mean ± SE (n = 6). Significant differences between WT and *ndt1$^-$:ndt1$^-$* plants, using Student's t-test, are indicated by asterisks * ($P < 0.05$). Scale bar: 100 micrômetro.
