# Supplementary material for: The mitochondrial NAD + transporter (NDT1) plays important roles in cellular NAD + homeostasis in Arabidopsis thaliana
Source: Plant J. 2019 Aug 9;100(3):487–504. doi: 10.1111/tpj.14452 (PMC6900047; doi:10.1111/tpj.14452)
Supplement: Supplementary file 9 — Figure S9. eFP display of transcript accumulation patterns across a variety of Arabidopsis organs and treatments. [file TPJ-100-487-s009.pdf]

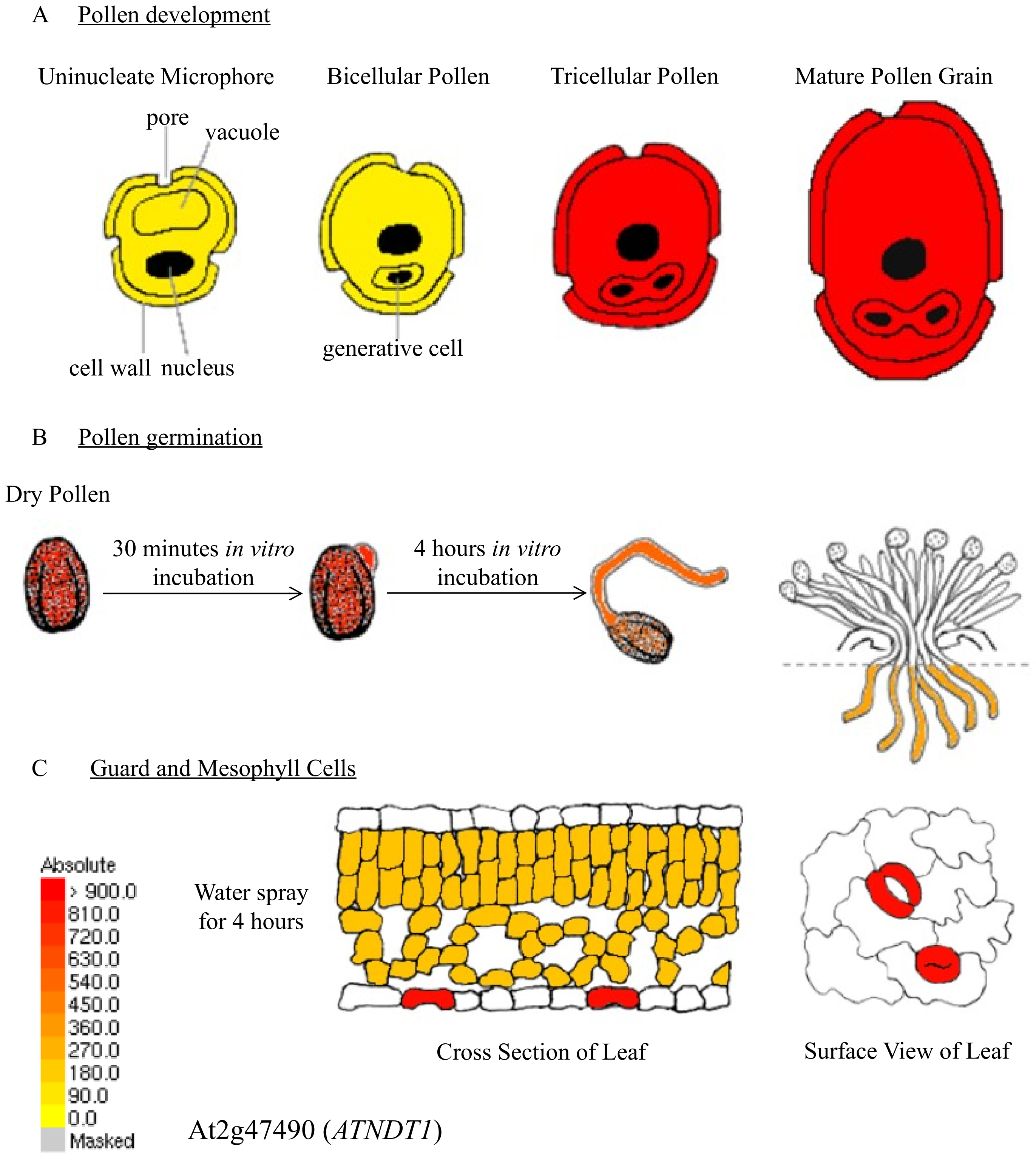

Figure S9. eFP display of transcript accumulation patterns across a variety of *Arabidopsis* organs and treatments. The images were obtained from the Bio-Array Resource for Arabidopsis Functional Genomics website (http://bar.utoronto.ca). Arabidopsis eFP browser presents the transcript accumulation pattern of At2g47490 during pollen development (A) and germination (B) and in guard and mesophyll cells (C). In all cases, red indicates higher level of transcript accumulation and yellow indicates a lower level of transcript accumulation.
