# Supplementary material for: The mitochondrial NAD + transporter (NDT1) plays important roles in cellular NAD + homeostasis in Arabidopsis thaliana
Source: Plant J. 2019 Aug 9;100(3):487–504. doi: 10.1111/tpj.14452 (PMC6900047; doi:10.1111/tpj.14452)
Supplement: Supplementary file 10 — Figure S10. Gas‐exchange and chlorophyll a fluorescence parameters in leaves of 4‐week‐old Arabidopsis thaliana genotypes deficient in the expression of the mitochondrial NAD+ transporter (NDT1) and wild type (WT) plants. [file TPJ-100-487-s010.pdf]

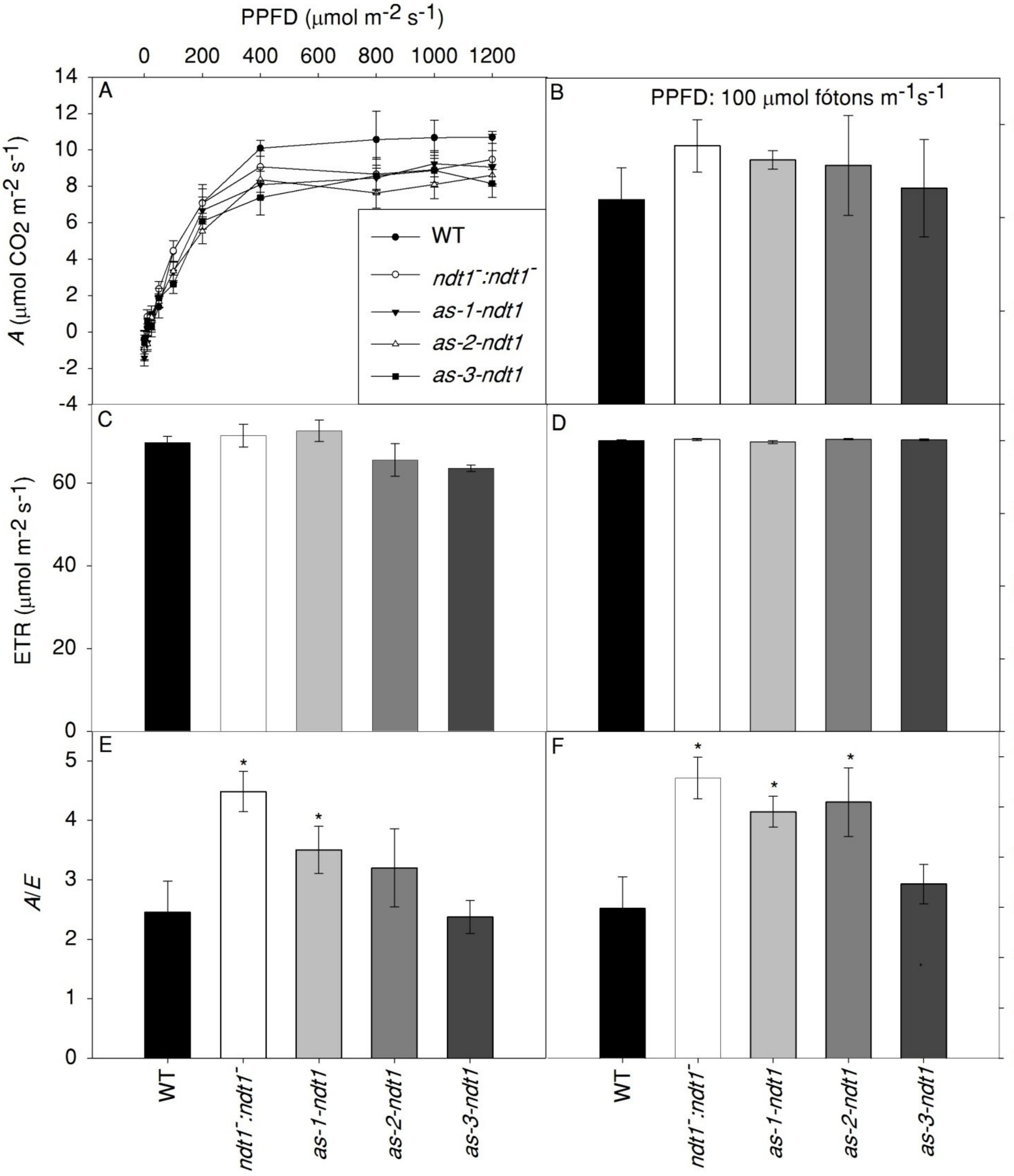

Figure S10. Gas exchange and chlorophyll *a* fluorescence parameters in leaves of 4-week-old *Arabidopsis thaliana* genotypes deficient in the expression of the mitochondrial NAD$^+$ transporter (NDT1) and wild type (WT) plants. (A) Light-response curve to changes in radiation photosynthetically active (RFA). (B) Assimilation rate (*A*) at 100 μmol m^{-2}s^{-1}. (C) Electron transport rate (ETR). (D) Photochemical efficiency of photosystem II (F_v/F_m). (E) Instantaneous water-use efficiency (*A*/*E*). (F) Intrinsic water-use efficiency (A/g_s). Values are presented as mean ± SE of six individual plants per line; an asterisk indicates values that were determined by Student's *t* test to be significantly different ($P < 0.05$) from the WT.
