# Supplementary material for: The mitochondrial NAD + transporter (NDT1) plays important roles in cellular NAD + homeostasis in Arabidopsis thaliana
Source: Plant J. 2019 Aug 9;100(3):487–504. doi: 10.1111/tpj.14452 (PMC6900047; doi:10.1111/tpj.14452)
Supplement: Supplementary file 12 — Figure S12. Changes in the main nitrogen metabolites in leaves of 4‐week‐old Arabidopsis thaliana genotypes deficient in the expression of the mitochondrial NAD+ transporter (NDT1) and wild type (WT) plants. [file TPJ-100-487-s012.pdf]

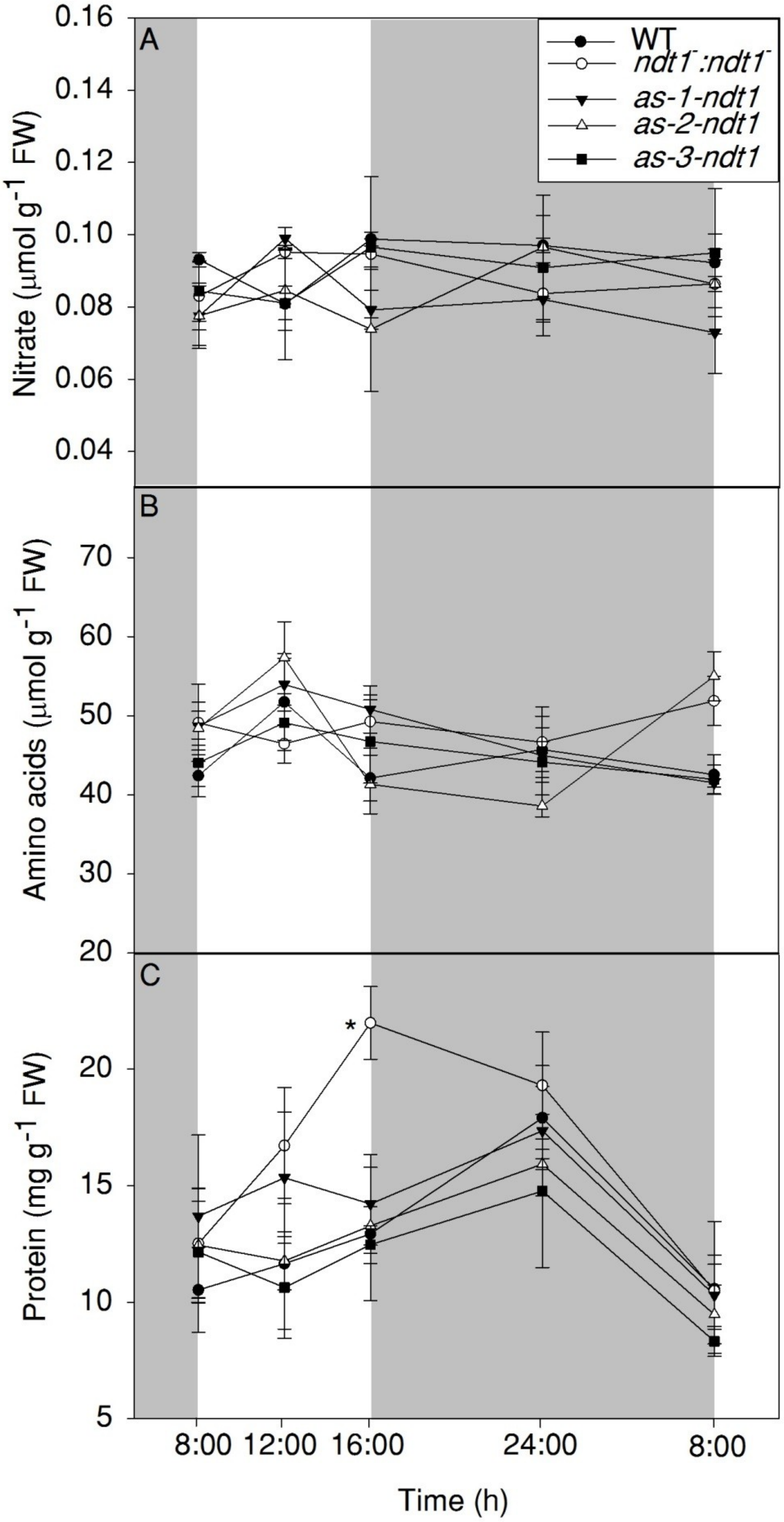

Figure S12. Changes in the main nitrogen metabolites in leaves of 4-week-old *Arabidopsis thaliana* genotypes deficient in the expression of the mitochondrial NAD$^+$ transporter (NDT1) and wild type (WT) plants. Values are presented as mean ± SE of six individual plants per line; an asterisk indicates values that were determined by Student's *t* test to be significantly different ($P < 0.05$) from the WT. Gray areas represent the dark period. FW: fresh weight.
