# Supplementary material for: The mitochondrial NAD + transporter (NDT1) plays important roles in cellular NAD + homeostasis in Arabidopsis thaliana
Source: Plant J. 2019 Aug 9;100(3):487–504. doi: 10.1111/tpj.14452 (PMC6900047; doi:10.1111/tpj.14452)
Supplement: Supplementary file 14 — Table S1. Parameters derived from photosynthetic light curve response (Figure S4a) of 4‐week‐old, short‐day grown Arabidopsis thaliana genotypes deficient in the expression of the mitochondrial NAD+ transporter (NDT1). [file TPJ-100-487-s014.docx]

**Supplementary table 1.** Parameters derived from photosynthetic light curve response (Figure S4A) of 4-week-old, short day grown *Arabidopsis thaliana* genotypes deficient in the expression of the mitochondrial NAD^+^ transporter (NDT1).

| Parameter | WT | *ndt1^-^:ndt1^-^* | *anti-1-ndt1* | *anti-2-ndt1* | *anti-3-ndt1* |
| --- | --- | --- | --- | --- | --- |
| *A*_RFA_ | 10.1 ± 0.5 | 9.1 ± 0.9 | 8.1 ± 0.7 | 8.4 ± 0.7 | 8.3 ± 0.9 |
| *I*_c_ | 21.3 ± 4.3 | 21.3 ± 4.0 | 22.0 ± 0.5 | 23.0 ± 1.9 | 16.9 ± 1.4 |
| *I*_s_ | 328 ± 42 | 336 ± 99 | 338 ± 54 | 321 ± 45 | 342 ± 40 |
| 1/ɸ | 18.5 ± 3.5 | 21.6 ± 4.0 | 24.5 ± 5.9 | 25.3 ± 9.4 | 23.4 ± 5.0 |

Values are presented as mean ± SE of determinations on six individual plants per line; an asterisk indicates values that were determined by Student’s *t* test to be significantly different (P < 0.05) from the WT. Abbreviations: *A*_RFA_: assimilation rate saturated by light (µmol CO_2_ m^-2^ s^-1^), *I*_c_: compensation irradiance (µmol m^-2^ s^-1^), *I*_s_: saturation irradiance (µmol m^-2^ s^-1^), 1/ɸ: light efficient use (µmol photons mol^-1^ CO_2_).
