# Supplementary material for: The mitochondrial NAD + transporter (NDT1) plays important roles in cellular NAD + homeostasis in Arabidopsis thaliana
Source: Plant J. 2019 Aug 9;100(3):487–504. doi: 10.1111/tpj.14452 (PMC6900047; doi:10.1111/tpj.14452)
Supplement: Supplementary file 15 — Table S2. Relative metabolite levels in leaves of 4‐week‐old, short‐day grown, Arabidopsis thaliana genotypes deficient in the expression of the mitochondrial NAD+ transporter (NDT1) and wild type (WT) plants. [file TPJ-100-487-s015.docx]

**Supplementary table 2.** Relative metabolite levels in leaves of 4-week-old, short day grown, *Arabidopsis thaliana* genotypes deficient in the expression of the mitochondrial NAD^+^ transporter (NDT1) and wild type (WT) plants.

| **Metabolites** | **WT** | ***ndt1^-^:ndt1^-^*** | ***as-1-ndt1*** | ***as-2-ndt1*** | ***as-3-ndt1*** |
| --- | --- | --- | --- | --- | --- |
| ***Amino acids*** |  |  |  |  |  |
| β-Alanine | 1.00 ± 0.06 | 0.92 ± 0.06 | 0.80 ± 0.05 | 1.03 ± 0.05 | 1.07 ± 0.07 |
| Asparagine | 1.00 ± 0.11 | 0.89 ± 0.37 | 0.87 ± 0.16 | 0.77 ± 0.12 | 0.88 ± 0.02 |
| Aspartate | 1.00 ± 0.07 | 0.84 ± 0.15 | 1.02 ± 0.17 | 0.84 ± 0.05 | 0.76 ± 0.07 |
| GABA | 1.00 ± 0.19 | 1.70 ± 0.65 | 1.44 ± 0.29 | 1.28 ± 0.34 | 1.24 ± 0.06 |
| Glutamate | 1.00 ± 0.46 | **3.24 ± 0.16** | **2.14 ± 0.14** | 1.17 ± 0.06 | 1.09 ± 0.17 |
| Glutamine | 1.00 ± 0.10 | 0.92 ± 0.16 | 0.92 ± 0.01 | 0.75 ± 0.08 | **0.49 ± 0.04** |
| Glycina | 1.00 ± 0.13 | 1.06 ± 0.46 | 0.94 ± 0.19 | 0.83 ± 0.04 | 0.84 ± 0.02 |
| Isoleucine | 1.00 ± 0.13 | 1.35 ± 0.78 | 1.33 ± 0.23 | 1.37 ± 0.21 | 1.32 ± 0.09 |
| Leucine | 1.00 ± 0.17 | **2.01 ± 0.70** | 1.51 ± 0.29 | 1.31 ± 0.20 | 1.37 ± 0.34 |
| Methionine | 1.00 ± 0.12 | 1.16 ± 0.56 | 1.12 ± 0.13 | 0.74 ± 0.15 | 0.88 ± 0.22 |
| Phenylalanine | 1.00 ± 0.15 | 1.26 ± 0.47 | 0.88 ± 0.08 | 1.05 ± 0.15 | 1.27 ± 0.12 |
| Proline | 1.00 ± 0.22 | 1.23 ± 0.67 | 0.99 ± 0.17 | 0.94 ± 0.16 | 0.90 ± 0.16 |
| Serine | 1.00 ± 0.12 | 1.20 ± 0.36 | 1.26 ± 0.13 | 1.04 ± 0.12 | 1.08 ± 0.20 |
| Threonine | 1.00 ± 0.12 | 1.01 ± 0.29 | 1.10 ± 0.26 | 1.07 ± 0.10 | 0.85 ± 0.10 |
| Tryptophan | 1.00 ± 0.21 | 1.12 ± 0.12 | 0.88 ± 0.12 | 0.99 ± 0.33 | 1.03 ± 0.10 |
| Tyrosine | 1.00 ± 0.35 | 1.14 ± 0.29 | 0.95 ± 0.12 | 1.09 ± 0.11 | 1.36 ± 0.20 |
| Valine | 1.00 ± 0.08 | 1.46 ± 0.23 | 0.28 ± 0.14 | 1.24 ± 0.13 | 1.00 ± 0.01 |
| ***Sugars*** |  |  |  |  |  |
| Fructose | 1.00 ± 0.30 | **2.43 ± 0.48** | 1.70 ± 0.52 | 1.44 ± 0.21 | **3.50 ± 0.73** |
| Glucose | 1.00 ± 0.08 | **3.26 ± 0.02** | **3.92 ± 0.06** | **2.23 ± 0.01** | **2.87 ± 0.13** |
| Maltose | 1.00 ± 0.18 | 1.15 ± 0.10 | 1.02 ± 0.11 | 0.91 ± 0.11 | 1.33 ± 0.17 |
| Raffinose | 1.00 ± 0.51 | 0.74 ± 0.45 | 0.47 ± 0.02 | 1.23 ± 0.36 | **2.89 ± 0.55** |
| Sorbose | 1.00 ± 0.78 | **2.06 ± 0.48** | 1.86 ± 0.43 | 1.08 ± 0.19 | 1.90 ± 0.42 |
| Sucrose | 1.00 ± 0.05 | 0.93 ± 0.05 | 0.89 ± 0.05 | 1.03 ± 0.02 | 1.08 ± 0.06 |
| Threalose, α-α- | 1.00 ± 0.25 | 1.04 ± 0.20 | 0.82 ± 0.07 | 1.08 ± 0.10 | **2.17 ± 0.08** |
| *Continues...* | | | | | |
|  | | | | | |
| **Metabolites** | **WT** | ***ndt1^-^:ndt1^-^*** | ***as-1-ndt1*** | ***as-2-ndt1*** | ***as-3-ndt1*** |
| ***Organic acids*** |  |  |  |  |  |
| Ascorbate | 1.00 ± 0.29 | **2.45 ± 0.85** | **1.78 ± 0.16** | 1.67 ± 0.41 | 1.99 ± 0.29 |
| Citrate | 1.00 ± 0.04 | 0.98 ± 0.15 | 1.15 ± 0.04 | 1.05 ± 0.06 | 1.13 ± 0.04 |
| Dehidroascorbate dimer | 1.00 ± 0.15 | 1.78 ± 0.20 | 1.69 ± 0.41 | 1.35 ± 0.17 | 1.07 ± 0.34 |
| Glycerate | 1.00 ± 0.06 | 1.20 ± 0.15 | 1.26 ± 0.16 | 1.20 ± 0.12 | 1.25 ± 0.09 |
| Glycolate | 1.00 ± 0.05 | 0.70 ± 0.17 | 0.79 ± 0.11 | 0.76 ± 0.07 | 0.86 ± 0.07 |
| Lactate | 1.00 ± 0.08 | 0.81 ± 0.10 | 0.92 ± 0.22 | 0.82 ± 0.06 | 1.10 ± 0.20 |
| Succinate | 1.00 ± 0.15 | 1.34 ± 0.25 | 0.64 ± 0.09 | 1.18 ± 0.06 | 1.22 ± 0.12 |
| Threonate | 1.00 ± 0.24 | 1.18 ± 0.24 | 1.15 ± 0.11 | 1.07 ± 0.23 | 1.36 ± 0.13 |
| ***Polyols*** |  |  |  |  |  |
| Erythritol | 1.00 ± 0.20 | **1.27 ± 0.10** | 0.86 ± 0.04 | 1.29 ± 0.12 | 1.15 ± 0.10 |
| Galatinol | 1.00 ± 0.40 | **2.94 ± 0.23** | 1.57 ± 0.13 | **2.26 ± 0.12** | **5.56 ± 0.78** |
| Myo-inositol | 1.00 ± 0.22 | **1.32 ± 0.16** | **0.46 ± 0.02** | 1.03 ± 0.21 | **1.43 ± 0.06** |
| ***Others*** |  |  |  |  |  |
| Putrescine | 1.00 ± 0.08 | 1.04 ± 0.50 | 0.81 ± 0.15 | 0.72 ± 0.09 | 0.78 ± 0.11 |
| Urea | 1.00 ± 0.08 | 1.14 ± 0.39 | 1.18 ± 0.24 | 1.08 ± 0.10 | 0.96 ± 0.01 |

Values are presented as mean ± SE of determinations on six individual plants per line; bold values indicates values that were determined by Student’s *t* test to be significantly different (*P* < 0.05) from the WT. GABA: γ-aminobutyric acid.
