# Supplementary material for: The mitochondrial NAD + transporter (NDT1) plays important roles in cellular NAD + homeostasis in Arabidopsis thaliana
Source: Plant J. 2019 Aug 9;100(3):487–504. doi: 10.1111/tpj.14452 (PMC6900047; doi:10.1111/tpj.14452)
Supplement: Supplementary file 16 — Table S3. List of primers used in this work to perform qPCR analysis. [file TPJ-100-487-s016.docx]

**Supplementary table 3.** List of primers used in this work to perform qPCR analysis.

| **Gene code** | **Description** | **Foward** | **Reverse** |
| --- | --- | --- | --- |
| AT2G39970 | PXN (Peroxissomal NAD transporter) | ACAACGTTACCGCTTTGGAGAC | TGACTGTAGCTCCGAGTTTCGC |
| At1g25380 | NDT2 (Mitochondrial NAD transporter) | CGATGCCATGTTCCAACTAC | CATCAAAAGGGCCAAAAAGT |
| At2g47490 | NDT1 (Mitochondrial NAD transporter) | GGGAATTCGCGGATTGTACAGTGG | TGGGAAACTGAATGGCAACATGAC |
| At5g14760 | Ao (Aspartate oxidase) | TGGTCGCTGGTGCTCATCTTTG | AGGCCCTTCAGTACACACAACTC |
| At5g50210 | Qs (Quinolinate synthase) | TAGCAGGTGGTGAAGGTTGCTC | AGCGAGCTAAGCGAGTTCATCTTC |
| At2g01350 | QPT (Quinolinic acid phosphoribosyl transferase) | TTGGGAAAGTATCAGGGAATGCAC | TGCAGCATCTGCCATTAACTTGG |
| At5g55810 | NMNAT (Nicotinate/nicotinamide mononucleotide adenyltransferase) | TGGCAACTGGGAGTTTCAATCCTC | TCTCTCGCCAGCTCAAACATGC |
| At1g55090 | NADS (NAD synthetase) | CAACAGCTGAGCTTGAGCCCATTC | CCATGTCGACTTCATCGAGCTGAG |
| At4g36940 | NAPRT1 (Nicotinate phosphoribosyltransferase 1) | AGAACGAACCACCTCCAAAGGTC | AGCTCTTCCCTAGCTTCATCTGC |
| At2g23420 | NAPRT2 (Nicotinate phosphoribosyltransferase 2) | AGTGCCACAACGTGTCGAAGAG | TTCTCTTGCTTCATCTGCACTTCC |
| At2G22570 | NIC1 (Nicotinamidase1) | CGGCAATATGGCTCCAACAAAGC | GCAAGCTTTGCACTTTCCTCCAC |
| At3g16190 | NIC4 (Nicotinamidase4) | ACTCGTCATCGACATGCAGAAC | CCTTTCACTTGCGTCACAGCAC |
| At4g12720 | NUDIX7 (Nudix hydrolase 7) | AATTCTCTCCAAGGTACACACAGC | CCATAGGTTCCACCATGGTTACAG |
| At2g31320 | PARP1 (Poly(ADP-ribose)polymerase1) | ATCGTCTACGATACAGCCCAGGTG | TGGTTCAGGCTCATCTCTTGTGC |
| At4g02390 | PARP2 (Poly(ADP-ribose)polymerase2) | ATGCTACTCTGGCACGGTTCAC | AGGAGGAGCTATTCGCAGACCTTG |
| At2g37620 | Actin | CTTGCACCAAGCAGCATGAA | CCGATCCAGACACTGTACTTCCTT |
| At3g21070 | NADK1 (NAD kinase 1) | GTCAATGGTCCATCCTCAGGTTCC | TGGGCGGAATGATAGAGAATGCG |
| At1G21640 | NADK2 (NAD kinase 2) | GGCAAAGCAATGCCTGGTAAAGTG | ACTCCATCGCCTTGTACCTTCG |
| At1G78590 | NADK3 (NAD kinase 3) | ACTCTGTTCCGGTTCTAGGAGTC | TGATCGCTCAGTTCTTCGACCTC |
| At4g27110 | COBL11 | CCCTGCTGCTACTTCTAAAGGC | TCAGGTCACCGTGTTGTCTAGG |
| At5g15710 | Fbox | TTTCGGCTGAGAGGTTCGAGT | GATTCCAAGACGTAAAGCAGATCAA |
