# Supplementary material for: The mitochondrial NAD + transporter (NDT1) plays important roles in cellular NAD + homeostasis in Arabidopsis thaliana
Source: Plant J. 2019 Aug 9;100(3):487–504. doi: 10.1111/tpj.14452 (PMC6900047; doi:10.1111/tpj.14452)
Supplement: Supplementary file 17 [file TPJ-100-487-s017.docx]

**LEGENDS FOR SUPPORTING INFORMATION**

**Figure S1. Gene expression analysis of the NDT1 gene in different organs of *Arabidopsis thaliana* wild type plants.** Relative transcript abundance was determined by qRT-PCR from samples of wild type plants in different stages of development (48 h imbibed seeds, 8 days-old seedlings, mature leaves from 28 days-old plants, closed (Day 0) and open (Day 4) flowers, and pollen grains. Relative abundance of transcripts was calculated as 2^-∆Ct^ by subtracting the CT of the targets for the CT of the housekeeping gene ACT2. The values shown are means of three repeats (indicated by error bars).

**Figure S2.** **Gene expression analysis of genes encoding NAD^+^ carriers (NDT1, NDT2 and PXN) in different organs of *Arabidopsis thaliana* wild type and ndt1^-^:ndt1^-^ plants.** The relative expression was determined by real-time quantitative PCR and calculated relative to the wild type (WT) in imbibed seeds, leaves from 28-days-old rosettes, open flowers (day 2) and pollen grains. Values are presented as mean ± SE of four individual plants per line; an asterisk indicates values that were determined by Student’s t test to be significantly different (*P* < 0.05) from the WT.

**Figure S3.** **Gene expression analysis of genes encoding enzymes related to NAD^+^ metabolism in imbibed seeds and leaves of *Arabidopsis thaliana* mutants deficient in the expression of the mitochondrial NAD^+^ transporter (NDT1) and wild type (WT) plants.** The relative expression levels of genes encoding NAD^+^ biosynthetic enzymes (A) and NAD^+^ kinases (B) were determined by quantitative real-time PCR. The values were calculated relative to the WT in seeds 48 h after imbibition and in rosette leaves of 28-days-old plants. Values are presented as mean ± SE of three individual plants per line; an asterisk indicates values that were determined by Student’s *t* test to be significantly different (*P* < 0.05) from the WT.

**Figure S4.** **Phenotypic analysis of *Arabidopsis thaliana* lines deficient in the expression of the mitochondrial NAD^+^ transporter (NDT1) and wild type (WT) plants.** (A) Silique width. (B) Seed width. (C) Weight of all plant seeds. Values are presented as mean ± SE of six individual plants per line; an asterisk indicates values that were determined by Student’s *t* test to be significantly different (*P* < 0.05) from the WT.

**Figure S5.** **Seed, seedling, germination and seedling establishment characterization of *Arabidopsis thaliana* mutant line deficient in the expression of the mitochondrial NAD^+^ transporter (NDT1) and wild type (WT) plants.** Values in figures A-D are presented as mean ± SE (n = 5) and in figures E-H are presented as mean ± SE of six individual plates with 50 seeds each per line; an asterisk indicates values that were determined by the Student’s *t* test to be signiﬁcantly different (*P* < 0.05) from the WT.

**Figure S6. Fatty acid composition in seeds and seedling of *Arabidopsis thaliana* mutants deficient in the expression of the mitochondrial NAD^+^ transporter (NDT1) and wild type (WT) plants.** Fatty acid composition was analyzed by GC of fatty acid methyl esters (FAMEs). (A) Seeds; (B) 2-days-old seedlings; (C) 4-days-old seedlings; (D) 6-days-old seedlings. Detail presenting FAMEs for best visualization. Values are presented as mean ± SE of three individual samples per line; an asterisk indicates values that were determined by the Student’s *t* test to be signiﬁcantly different (*P* < 0.05) from the WT.

**Figure S7. Phenotypic analysis of pollen grains stained with acetic carmine from *Arabidopsis thaliana* genotypes deficient in the expression of the mitochondrial NAD^+^ transporter (NDT1) and wild type plants.** Pollen grains stained with red were considered as viable and with yellow the non-viable ones (indicated with an arrow). Unstained or deformed pollen grains were also considered as non-viable. (A) Wild type (WT); (B) *ndt1^-^:ndt1^-^*; (C) *as-1-ndt1*; (D) *as-2-ndt1*; (E) *as-3-ndt1*; (F) detail of WT pollen grain; (G) detail *ndt1^-^:ndt1^-^* non-viable pollen grain (smaller than WT); (H) detail *as-2-ndt1* deformed pollen grain. Bars: 20 µm.

**Figure S8. Germination rate and tube growth of pollen grains from *Arabidopsis thaliana* genotype deficient in the expression of the mitochondrial NAD^+^ transporter (NDT1) and wild type (WT) plants.** (A) Percentage of pollen tube growth which represents germination/total number of grains analyzed. (B) Pollen tube length. Pollen grains from 3 flowers per replication were placed immediately onto solidified pollen-germination medium and incubated at 22ºC for 8 hours. Bars represent mean ± SE (n = 6). Significant differences between WT and *ndt1^-^:ndt1^-^* plants, using Student’s t-test, are indicated by asterisks * (*P* < 0.05). Scale bar: 100 micrômetro.

**Figure S9. eFP display of transcript accumulation patterns across a variety of Arabidopsis organs and treatments.** The images were obtained from the Bio-Array Resource for Arabidopsis Functional Genomics website ([http://bar.utoronto.ca](http://bar.utoronto.ca/)). Arabidopsis eFP browser presents the transcript accumulation pattern of At2g47490 during pollen development (A) and germination (B) and in guard and mesophyll cells (C). In all cases, red indicates higher level of transcript accumulation and yellow indicates a lower level of transcript accumulation.

**Figure S10. Gas exchange and chlorophyll *a* fluorescence parameters in leaves of 4-week-old *Arabidopsis thaliana* genotypes deficient in the expression of the mitochondrial NAD^+^ transporter (NDT1) and wild type (WT) plants.** (A) Light-response curve to changes in radiation photosynthetically active (RFA). (B) Assimilation rate (*A*) at 100 µmol m^-2^s^-1^. (C) Electron transport rate (ETR). (D) Photochemical efficiency of photosystem II (*F*_v_/*F*_m_). (E) Instantaneous water-use efficiency (*A*/*E*). (F) Intrinsic water-use efficiency (*A*/*g*_s_). Values are presented as mean ± SE of six individual plants per line; an asterisk indicates values that were determined by Student’s *t* test to be significantly different (*P* < 0.05) from the WT.

**Figure S11. Non-photochemical quenching (NPQ) of 4-week-old *Arabidopsis thaliana* genotypes deficient in the expression of the mitochondrial NAD^+^ transporter (NDT1) and wild type (WT) plants under various light intensities.** The data were obtained by light-response curve to changes in radiation photosynthetically active (RFA). Values are presented as mean ± SE of six individual plants per line. An asterisk indicates values that were determined by Student’s *t* test to be significantly different (*P* < 0.05) from the WT.

**Figure S12. Changes in the main nitrogen metabolites in leaves of 4-week-old *Arabidopsis thaliana* genotypes deficient in the expression of the mitochondrial NAD^+^ transporter (NDT1) and wild type (WT) plants.** Values are presented as mean ± SE of six individual plants per line; an asterisk indicates values that were determined by Student’s *t* test to be significantly different (*P* < 0.05) from the WT. Gray areas represent the dark period. FW: fresh weight.

**Figure S13. Changes in chlorophyll content in leaves of 4-week-old *Arabidopsis thaliana* genotypes deficient in the expression of the mitochondrial NAD^+^ transporter (NDT1) and wild type (WT) plants.** Values are presented as mean ± SE of six individual plants per line; an asterisk indicates values that were determined by Student’s *t* test to be significantly different (*P* < 0.05) from the WT. FW: fresh weight.

**Legends for supplementary tables**

**Supplementary table 1.** Parameters derived from photosynthetic light curve response (Figure S4A) of 4-week-old, short day grown *Arabidopsis thaliana* genotypes deficient in the expression of the mitochondrial NAD^+^ transporter (NDT1).

**Supplementary table 2.** Relative metabolite levels in leaves of 4-week-old, short day grown, *Arabidopsis thaliana* genotypes deficient in the expression of the mitochondrial NAD^+^ transporter (NDT1) and wild type (WT) plants.

**Supplementary table 3.** List of primers used in this work to perform qPCR analysis.
