# Supplementary material for: The mitochondrial NAD + transporter (NDT1) plays important roles in cellular NAD + homeostasis in Arabidopsis thaliana
Source: Plant J. 2019 Aug 9;100(3):487–504. doi: 10.1111/tpj.14452 (PMC6900047; doi:10.1111/tpj.14452)
Supplement: Supplementary file 3 — Figure S3. Gene expression analysis of genes encoding enzymes related to NAD+ metabolism in imbibed seeds and leaves of Arabidopsis thaliana mutants deficient in the expression of the mitochondrial NAD+ transporter (NDT1) and wild type (WT) plants. [file TPJ-100-487-s003.pdf]

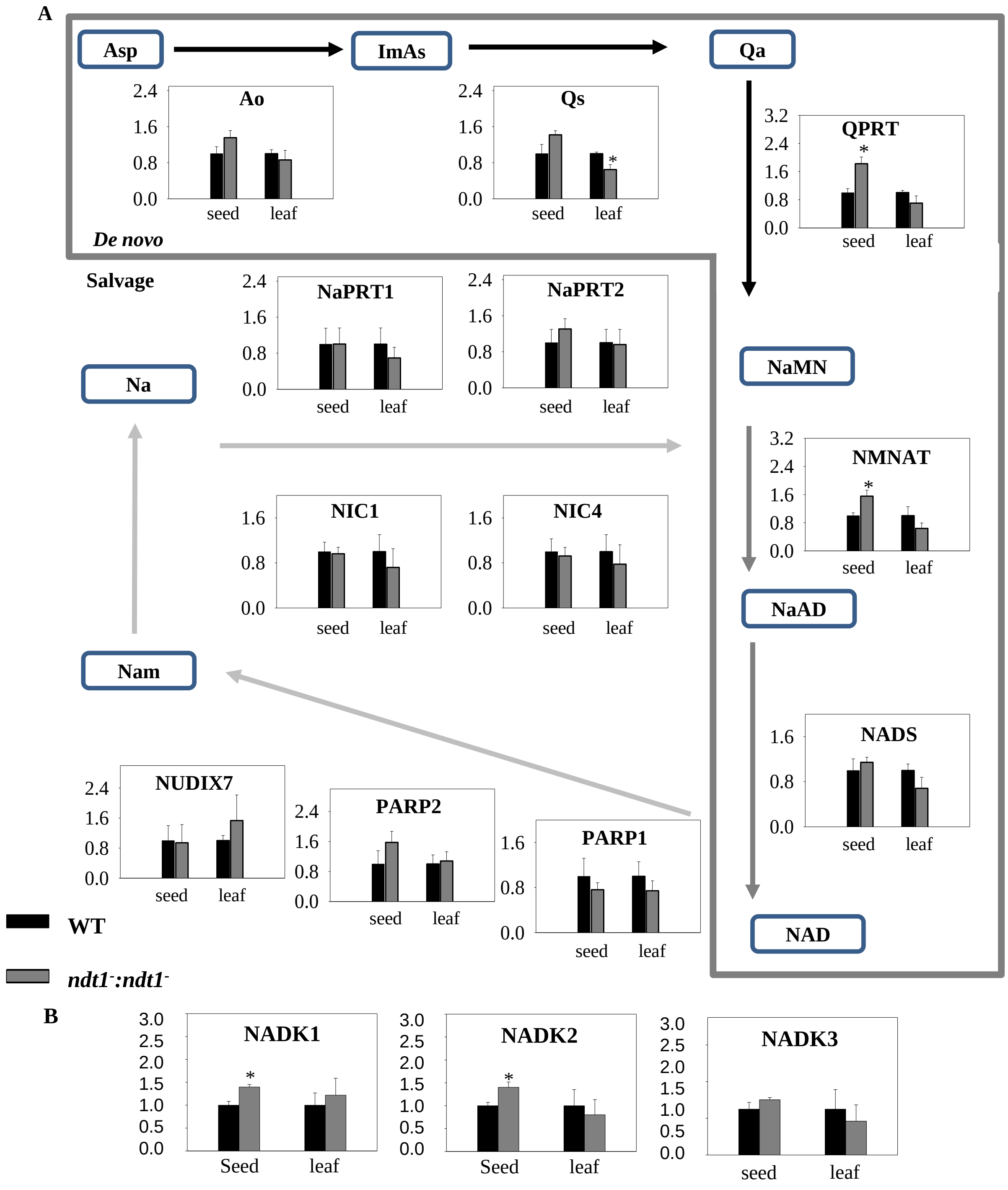

Figure S3. Gene expression analysis of genes encoding enzymes related to NAD$^+$ metabolism in imbibed seeds and leaves of *Arabidopsis thaliana* mutants deficient in the expression of the mitochondrial NAD$^+$ transporter (NDT1) and wild type (WT) plants.
The relative expression levels of genes encoding NAD$^+$ biosynthetic enzymes (A) and NAD$^+$ kinases (B) were determined by quantitative real-time PCR. The values were calculated relative to the WT in seeds 48 h after imbibition and in rosette leaves of 28-days-old plants. Values are presented as mean ± SE of three individual plants per line; an asterisk indicates values that were determined by Student's *t* test to be significantly different ($P < 0.05$) from the WT
